# Sex-specific lipid molecular signatures in obesity-associated metabolic dysfunctions revealed by lipidomic characterization in ob/ob mouse

**DOI:** 10.1186/s13293-019-0225-y

**Published:** 2019-02-26

**Authors:** Marcela González-Granillo, Luisa A. Helguero, Eliana Alves, Amena Archer, Christina Savva, Matteo Pedrelli, Osman Ahmed, Xidan Li, Maria Rosário Domingues, Paolo Parini, Jan-Åke Gustafsson, Marion Korach-André

**Affiliations:** 1 Department of Biosciences and Nutrition, Center for Innovative Medicine, Karolinska Institutet, Huddinge, Sweden; 2 Department of Medical Sciences, Institute for Biomedicine, University of Aveiro, Aveiro, Portugal; 3 Mass spectrometry Centre, Department of Chemistry (QOPNA, CESAM & ECOMARE), University of Aveiro, Aveiro, Portugal; 4 Division of Clinical Chemistry, Department of Laboratory Medicine, Karolinska Institutet at Karolinska University Hospital Huddinge, Huddinge, Sweden; 5 Department of Proteomics, Science for Life Laboratory, School of Biotechnology, KTH, Stockholm, Sweden; 6 Department of Biology and Biochemistry, Center for Nuclear Receptors and Cell Signalling, University of Houston, Houston, TX USA; 7 Department of Medicine, Metabolism and Molecular Nutrition Unit, Center for Endocrinology, Metabolism and Diabetes, Karolinska Institutet, S-141 86 Stockholm, Sweden; 8 Department of Medicine, Karolinska Institutet/AstraZeneca Integrated Cardio Metabolic Center, Karolinska Institutet at Karolinska University Hospital Huddinge, C2-94, S-141 86 Stockholm, Sweden

**Keywords:** Lipidomics, Fatty acids, Obesity, Metabolic syndrome, Sex

## Abstract

The response to overfeeding is sex dependent, and metabolic syndrome is more likely associated to obesity in men or postmenopausal women than in young fertile women. We hypothesized that obesity-induced metabolic syndrome is sex dependent due to a sex-specific regulation of the fatty acid (FA) synthesis pathways in liver and white adipose depots. We aimed to identify distinctive molecular signatures between sexes using a lipidomics approach to characterize lipid species in liver, perigonadal adipose tissue, and inguinal adipose tissue and correlate them to the physiopathological responses observed. Males had less total fat but lower subcutaneous on visceral fat ratio together with higher liver weight and higher liver and serum triglyceride (TG) levels. Males were insulin resistant compared to females. Fatty acid (FA) and TG profiles differed between sexes in both fat pads, with longer chain FAs and TGs in males compared to that in females. Remarkably, hepatic phospholipid composition was sex dependent with more abundant lipotoxic FAs in males than in females. This may contribute to the sexual dimorphism in response to obesity towards more metaflammation in males. Our work presents an exhaustive novel description of a sex-specific lipid signature in the pathophysiology of metabolic disorders associated with obesity in *ob*/*ob* mice. These data could settle the basis for future pharmacological treatment in obesity.

**Electronic supplementary material:**

The online version of this article (10.1186/s13293-019-0225-y) contains supplementary material, which is available to authorized users.

## Introduction

The liver is the main site for endogenous synthesis of fatty acids (FAs), and the adipose tissue (AT) is a major storage depot for excess lipids. In obesity, imbalance between energy intake and energy expenditure leads to storage of ectopic fat, as triglycerides (TGs) in non-adipose tissues including liver. Dysregulation of hepatic de novo lipogenesis (DNL) is a common feature of obesity and obesity-associated metabolic diseases such as insulin resistance (IR) and non-alcoholic fatty liver (NAFL). Hence, it is not surprising that extensive efforts have been committed to understand the link between obesity and these diseases. Fat metabolism is regulated by DNL/lipid uptake and lipolysis/oxidation of TGs and FAs. During a period of excess food intake, there is an imbalance between these two systems that drives towards more fat storage. Subcutaneous adipose tissue (SAT) acts as a powerful metabolic sink for FAs and TGs but, as adipocytes grow larger, they become dysfunctional and release FA metabolites that are the major cause of lipotoxicity and inflammation [1]. IR is associated with an increased adipocyte lipolysis with abundant circulating free FAs [2]. In addition, free FAs cause changes in membrane fluidity and availability of cell signaling molecules due to their effects on the lipid bilayer phospholipid (PL) molecular species composition [3]. In obese mice, lipid accumulation and alteration of PL composition promote IR [4] being many PL molecular species pro- or anti-inflammatory [5].

Despite the numerous metabolic studies on obesity, sex specificity during obesity has been poorly investigated. Clinical studies showed that women are more responsive to stimuli that increase the drive to eat [6], more affected by obesity, and more resistant to weight loss [7]. In humans, sexual dimorphism has been described not only related to body weight control, but also to body composition, fat distribution, and fuel metabolism [8–10]. Therefore, a sex-specific regulation on the expression of genes involved in lipid metabolism pathways is likely to occur. Interestingly, while obesity is more prevalent in women than in men [7], the latter are more prone to metabolic disorders [11]. Visceral adipose tissue (VAT) is increased in males and is considered a more metabolically deleterious AT depot than SAT [12]. The type of adipocytes, their endocrine function, lipolytic activity, response to insulin, and other hormones differ between the two fat depots.

We aimed to identify whether there is sexual dimorphism in lipid metabolism. For this purpose, we used the *ob*/*ob* mouse, a well-recognized model of human obesity [13]. To better understand sex-dependent FA synthesis pathways leading to obesity, we aimed to identify distinctive molecular signatures between genders, using a lipidomics approach, to characterize lipid species in liver, perigonadal visceral adipose tissue (gAT), and subcutaneous inguinal adipose tissue (iAT), and to correlate them to the physio pathological responses observed. The use of *ob*/*ob* mouse model was motivated by the possibility to induce obesity on a chow diet where most of the differences in the lipid species found by lipidomic analysis are the result of de novo FA synthesis.

## Research design and methods

### Animals

Weight-matched 7–8-week-old *ob*/*ob* (B6.V-Lep^ob^/J) female (F) and male (M) mice and, C57Bl/6J wild-type (WT) F and M mice, F estrogen receptor (ER) α knockout (ERαKO), and ERβ knockout (ERβKO) [14] mice were maintained in a temperature-controlled 12-h light/darkroom with free access to water and chow diet (R34, Lantmännen, Lantbruk, Sweden) or high-fat diet (HFD, research diet D12492). C57Bl/6 M mice have very low level of testosterone [15]; therefore, we used C57Bl/6 M mice to avoid to castrate males and stay in physiological conditions. Half of the WT M on HFD were treated IP, every other day, with estrogen (E2, 0.05 mg/kg body weight) for 3 weeks. For the *ob*/*ob* mice group, food intake was measured twice a week over the 5-week experimental period. Mice were anesthetised with 4% isoflurane at 9 AM, blood was immediately collected by heart puncture, and mice were euthanised by cervical dislocation. Liver, gAT (as a representative of visceral adipose tissue (VAT)), and iAT (as a representative of subcutaneous adipose tissue (SAT)) were collected and fixed in paraformaldehyde (PFA) or immediately frozen in liquid nitrogen for further analysis. The local Ethical Committee of the Swedish National Board of Animal Research approved all experiments.

### Magnetic resonance imaging/body adiposity

Total body fat mass and lean mass were assessed using magnetic resonance imaging system (EchoMRI). Unanesthetized mice were placed in a restraint tube and inserted into the EchoMRI system. At killing, liver and individual fat pads were weighed.

### Tolerance tests

Mice were fasted for 6 h prior to the glucose test and 4 h prior to the insulin test (*n* = 7 per sex). The tests were completed as explained in previous publications [16] and Homa-IR calculated as previously described [17]. Matsuda index and direct measurement of hepatic insulin sensitivity (ISI) have been calculated as described [18, 19]. Briefly, the Matsuda index was calculated as follows: Matsuda index = 1000/(√[*G*_0_ × *I*_0_ × *G*_mean_ × *I*_mean_]), the suffix *mean* indicates the average value of glucose and insulin concentration measured during the whole length of the test. Hepatic insulin sensitivity index was calculated as ISI = *k*/(FPG × FPI).

### Quantitative PCR

Total RNA was extracted using TRIzol (Invitrogen AB) and mRNA expression levels were quantified as described [16] and normalized to female group. Relative gene expression changes were calculated using 36b4 gene for adipose tissue and Tf2b and β-actin for liver as internal references. List of the primers used for RT-PCR and their sequence can be find in Supplementary Table S3 (Additional file 1).

### Histology and immunohistochemistry

Liver, gAT, and iAT were dissected, fixed in PFA, and embedded in paraffin [20]. Sections (4 μm thickness) were stained with hematoxylin-eosin (H&E), Adipophilin (ADRP, PROGEN Biotechnik, Germany) and F4/80 (ab6640, Abcam) were detected according to standard histological procedures. The number of crown-like structures was determined from the mean value calculated on five different fields of one section for each animal, using a magnification of × 2 to cover the whole region.

### Biochemical analysis of serum and liver

After blood collection, serum was aliquoted and stored at − 80 °C. ELISA kits were used to measure insulin (#EZRMI-13 K, Millipore), FGF21 (#MF2100, R&D systems), resistin (#MRSN00, R&D systems), and adiponectin (#MRP300, R&D systems) levels. For the measurement of the inflammatory cytokines in serum, a Bio-Plex Pro™ Mouse Cytokine Th17 Panel A 6-Plex was used (#M60-00007NY). Serum and liver TGs were measured by enzymatic assay using commercially available kits (Roche Diagnostics GmbH, Mannheim and mti Diagnostic GmbH, Idstein, Germany). The hepatic TG levels were corrected for the hepatic protein content, measured according to the Lowry method in the tissues digested with NaOH (1 M).

### Lipidomics analysis

TG content in gAT and iAT was quantified from total lipid extracts [21, 22] using the colorimetric kit LiquickCor-TG (Cormay) and related to tissue weight. TG fractions were recovered from total lipid extracts by solid phase extraction and analyzed by electrospray ionization mass spectrometry (ESI-MS) and MS/MSinaQ-ToF2 (Micromass) [20]. In liver, phospholipid (PL) amounts were estimated from total phosphorus content [23]. Identification of PL molecular species was carried out by LC-MS/MS analysis as in [24]. The degree of saturation of the corresponding FAs was confirmed by analysis of FA methyl esters (FAME) obtained by transmethylation of the total lipid extract using gas chromatography with flame ionization detector (GC-FID) [25]. Analysis was carried out in triplicate in at least three samples from each group.

### Calculations

Desaturase, elongase, and lipogenic activities were estimated from the product-to-precursor ratios of the percentage of individual FAs according to the following equations: Δ9 desaturation index = (C18:1 + C16:1)/(C18:0 + C16:0), Δ5 desaturation index = C18:2/C18:1, elongase activity index = C18:0/C16:0, SCD1 activity index = C18:1/C18:0, and the lipogenic index = C16:0/C18:2 as described [26, 27].

### Unsupervised clustering

The between-sample normalization with TMM method [28] was performed to sample matrix, where each sample is as the column and each physical parameter is as the row. Unsupervised clustering was then performed to the normalized matrix by *t-*SNE plot with the R package Rtsne [29]. The *t-*SNE is based on the most 50 variant dimensions of initial PCA plot. The speed/accuracy trade-off was set as 0.0 for the exact *t-*SNE distance matrix. The perplexity is set to 1 with optimal clusters shape. Plots showing all samples are based on the *t*-SNE field parameters V1 and V2 [29].

### Statistical analysis

Values are expressed as mean ± sem. Differences between groups were determined by multiple *t* tests corrected for multiple comparison. Statistical significance was determined using the Holm-Sidak method, with alpha = 5.000% in GraphPad Prism (San Diego, CA). Each row was analyzed individually, without assuming a consistent SD. *, *P* < 0.05, **, *P* < 0.01, ***, *P* < 0.001 and ****, *P* < 0.0001 M *vs* F; ^#^, *P* < 0.05, ^##^, *P* < 0.01, ^###^, *P* < 0.001 and ^####^, *P* < 0.0001 M HFD *vs* M HFD-E2 and ^&^, *P* < 0.05, ^&&^, *P* < 0.01, ^&&&^, *P* < 0.001 and ^&&&&^, *P* < 0.0001, F WT *vs* F ERKO were considered significant.

## Results

### Sex-dependent fat distribution and metabolic response to weight gain

**Fig. 1:**
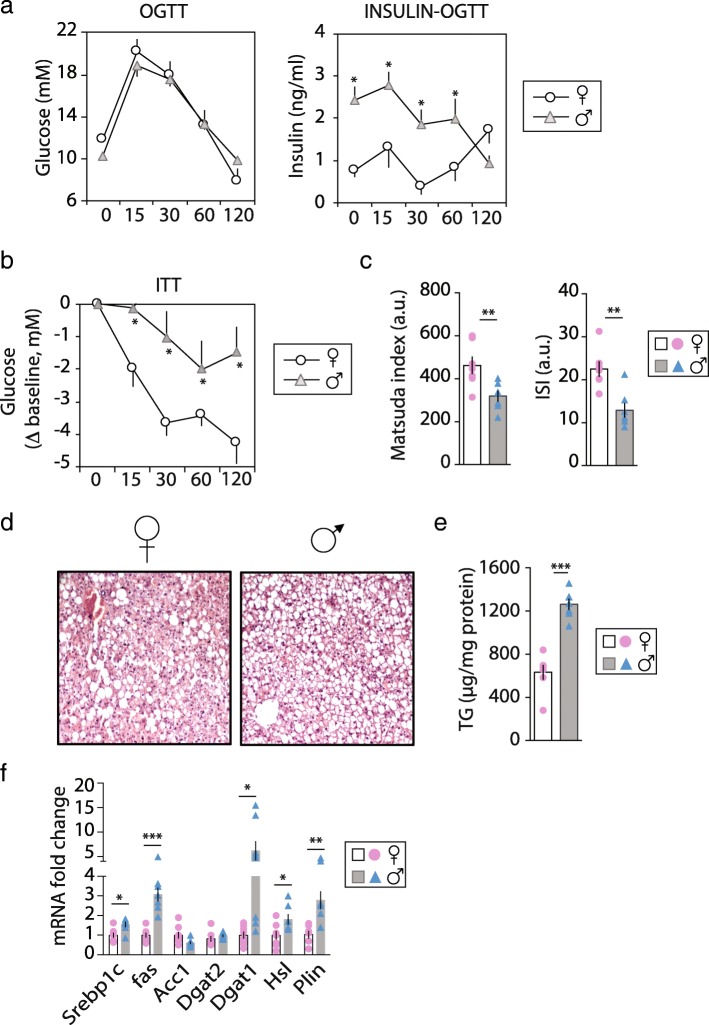
Sex-dependent fat distribution and metabolic response to weight gain. *Ob/ob* female (F) (♀ open bars and pink bullets) and male (M) (♂ gray bars and blue triangles) mice fed a chow diet for 5 weeks. Blood (**a**) glucose and serum insulin levels during the oral glucose tolerance test (OGTT); **b** delta blood glucose level from baseline (T0) during the insulin tolerance test (ITT); **c** matsuda index was used as a measure of whole body insulin sensitivity and ISI as an index of hepatic insulin sensitivity (*n* = 7); **d** representative hepatic histological sections stained for hematoxylin and eosin (*n* = 4); **e** liver triglycerides (TG) level (*n* = 7); **f** expression levels of de novo lipogenesis (*Srebp1c*, *Fas Acc1)* and triglycerides synthesis (*Dgat1* and *Dgat2*) genes. Values are mean ± sem, *n* = 6–9; (*P* < 0.05)*, M vs F. Abbreviations: *Srebp* sterol regulatory binding transcription factor, *Fas* fatty acid synthase, *Acc1* acetyl-CoA carboxylase, *Dgat* diacylglycerol O-acyltransferase, *Hsl* hormone sensitive lipase and *Plin* perilipin

Chow-fed F and M *ob*/*ob* mice had similar body weight (BW) gain and food intake, but M had a lower percentage of fat mass (54% vs 57%, *p* < 0.01) and higher lean mass (42% vs 38%, *p* < 0.01) than F. Fat distribution differed between sexes with M accumulating more VAT and less SAT than F (Table 1 and Additional file 2: Figure S1A-B). F and M showed similar serum fasting glucose level but fasting insulin level was 60% higher in M (Table 1). To evaluate the ability of the whole body to glucose clearance, we challenged the mice with a glucose load given by gavage (OGTT). Glucose level in the circulation was similar between M and F; however, insulin level during the OGTT was significantly higher in M compared t F at all time points, except for time 120 (Fig. 1a). In line with these results, glucose uptake in response to insulin injection was higher in F than M (Fig. 1b). The Matsuda index and hepatic insulin sensitivity index were higher in F together with lower Homa-IR (Fig. 1c and Table 1).

**Table 1:** Body weight, body adiposity, lean body mass, and serum analysis

	F	M		*P* values
Final BW (g)	47.6 ± 1.1	49.8 ± 0.7		ns
BW gain (g)	7.9 ± 0.8	8.1 ± 0.8		ns
Total body fat mass (g)	28.3 ± 0.5	26.1 ± 0.6	*	*P* < 0.05
Lean mass (g)	19.0 ± 0.4	20.3 ± 0.4	*	*P* < 0.05
% fat mass	57 ± 1	54 ± 1	**	*P* < 0.01
% lean mass	38 ± 1	42 ± 1	**	*P* < 0.01
Cumulative food intake (g)	1217 ± 1	1255 ± 1		ns
Food efficiency (g/ΔBW)	154 ± 12	155 ± 10		ns
Liver (%TF)	8.5 ± 0.3	11.5 ± 0.2	***	*P* < 0.001
VAT (%TF)	27.1 ± 0.5	29.3 ± 0.6	*	*P* < 0.05
SAT (%TF)	20.8 ± 0.3	17.0 ± 0.9	**	*P* < 0.01
SAT/VAT	0.77 ± 0.02	0.58 ± 0.03	***	*P* < 0.001
Serum analysis				
Fasted glucose (mM)	10.7 ± 0.3	9.1 ± 0.4		ns
Fasted insulin (ng/ml)	2.1 ± 0.2	3.4 ± 0.4	*	*P* < 0.05
HOMA-IR	0.8 ± 0.1	1.3 ± 0.1	**	*P* < 0.01
Triglycerides (mM)	0.20 ± 0.01	0.65 ± 0.09	**	*P* < 0.01
Adiponectin (pg/ml)	5740 ± 361	4985 ± 212		*P* = 0.06
Resistin (pg/ml)	22.6 ± 2.9	44.6 ± 2.4	**	*P* < 0.01
FGF21 (pg/ml)	5380 ± 189	3583 ± 444	**	*P* < 0.01
TNFα (pg/ml)	465 ± 72	374 ± 55		ns
IL1β (pg/ml)	88 ± 17	94 ± 16		ns
IL6 (pg/ml)	29 ± 7	21 ± 3		ns
IL10 (pg/ml)	437 ± 83	239 ± 28	*	*P* = 0.01

Data are presented as mean ± sem, *n* = 7–10. Differences between sexes were determined by two-tailed student’s *t* test with statistical significance determined using the Holm-Sidak method, with alpha = 5.000%. Each row was analyzed individually, without assuming a consistent SD. *, *P* < 0.05, **, *P* < 0.01, ***, *P* < 0.001 M vs F were considered significant. ns = not significant. *M* male, *F* female, *TF* total fat, *VAT* visceral fat (comprises the gAT, omental and retroperitoneal adipose depots), *SAT* subcutaneous adipose tissue (comprises the iAT and dorsal adipose depots), *HOMA-IR* homeostatic model assessment-insulin resistance, *FGF21* fibroblast growth factor 21, *TNF* tumor necrosis factor and *IL* interleukin

The H&E staining and adipophylin immunostaining of the F and M livers revealed that both sexes showed lipid droplets accumulation; however, M livers showed more lipid droplet accumulation and a higher level of hepatic TGs than F (Fig. 1d–e and Additional file 2: Figure S1). In addition, liver weight and serum TGs were significantly higher in M (Table 1). Serum levels of insulin-sensitized adipokines, FGF21, and adiponectin were higher in F, whereas the level of resistin was 2.5-fold higher in M (Table 1). Hepatic mRNA levels of DNL genes *Srepb1c* and *Fas* were higher in M (Fig. 1f), and mRNA levels of *Dgat*2, which coordinates the last step of newly synthetized TGs, was similar between sexes. However, the expression level of *Dgat1*, involved in the re-esterification of diacylglycerol into TGs, was 6 times higher in M than F. Finally, expression levels of *Plin*, a cytosolic lipid droplet-coated protein, and hormone sensitive lipase (*Hsl*) were higher in M than F (Fig. 1f). These data are in line with the higher liver TG content in M compared to F. Taken together, these findings imply that, despite a higher fat mass, F displayed enhanced insulin sensitivity, associated with an improved lipid metabolic profile compared to M.

### Sex-dependent inflammatory response to obesity in liver, gAT, and iAT

**Fig. 2:**
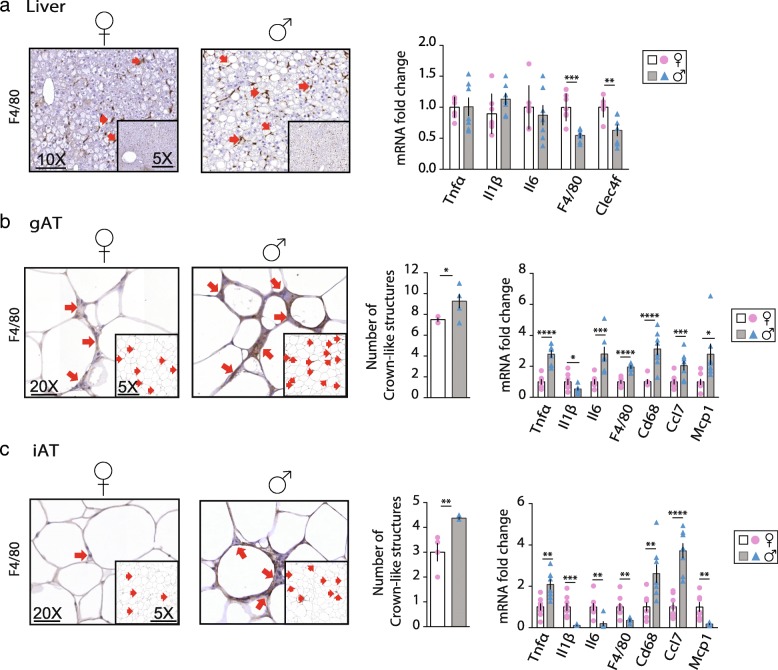
Sex-dependent inflammatory response to obesity in liver, gAT and iAT. Representative histological sections of staining for F4/80 (*n* = 4) and relative mRNA expression level of inflammatory genes in **a** liver **b** gAT and **c** iAT; and **b** gAT and **c** iAT quantification of crown like structures in F (♀ - open bars and pink bullets) and M (♂ gray bars and blue triangles) mice (*n* = 7). Values are mean ± sem, *n* = 6–9; *, P < 0.05, **, *P* < 0.01, ***, *P* < 0.001 and ****, *P* < 0.0001 M vs F were considered significant. *Tnfα* tumor necrosis factor alfa, *Il* interleukin*, Clec4f* C-type lectin domain family 4 member F, *Cd68* cluster of differentiation 68*, Ccl* chemokine (C-C motif) ligand and *Mcp* Monocyte chemoattractant protein

Results from Fig. 1 described a sex-dependent metabolic response to obesity, and lipid accumulation may result in an increased production of inflammatory mediators generated from FAs. Therefore, histological sections of F and M liver, gAT, and iAT were immunostained for F4/80, indicative of macrophage infiltration. Liver sections did not show differences between sexes in F4/80 positive staining (Fig. 2a). However, hepatic expression level of pro-inflammatory genes *F4*/*80* and *Clec4f* were higher in F than in M. In gAT, adipocyte size was similar between sexes but the number of crown-like structures was higher in M together with a higher expression level of the pro-inflammatory genes *Tnf*-*α*, *Il6*, *F4*/*80*, *Cd68*, *Ccl7*, and *Mcp1* except for *Il1*-*β* that was lower (Fig. 2b). In iAT, M showed a higher amount of crown-like structures together with a higher expression level of *Tnf*-*α*, *Cd68*, and *Ccl7* and lower expression level of *Il1*-*β*, *Il6*, *F4*/*80,* and *Mcp1* compared to F (Fig. 2c), and no differences between adipocyte sizes were observed. Circulating levels of pro-inflammatory cytokines (i.e., TNFα, IL1β and IL6) were similar between sexes. However, it is important to note that the serum level of the anti-inflammatory cytokine IL10 was 2.5-times higher in the F than in M (Table 1).

To conclude, F tend to present pro-inflammatory markers in liver despite a much less steatotic liver compared to M. In contrast, M are more prone to develop inflammation in gAT despite lower total fat content as compared to F. Together, our data show that each sex presents differential susceptibility to obesity-induced inflammation accompanied with a tissue-specific response.

### Sex-dependent FA profile in liver

**Fig. 3:**
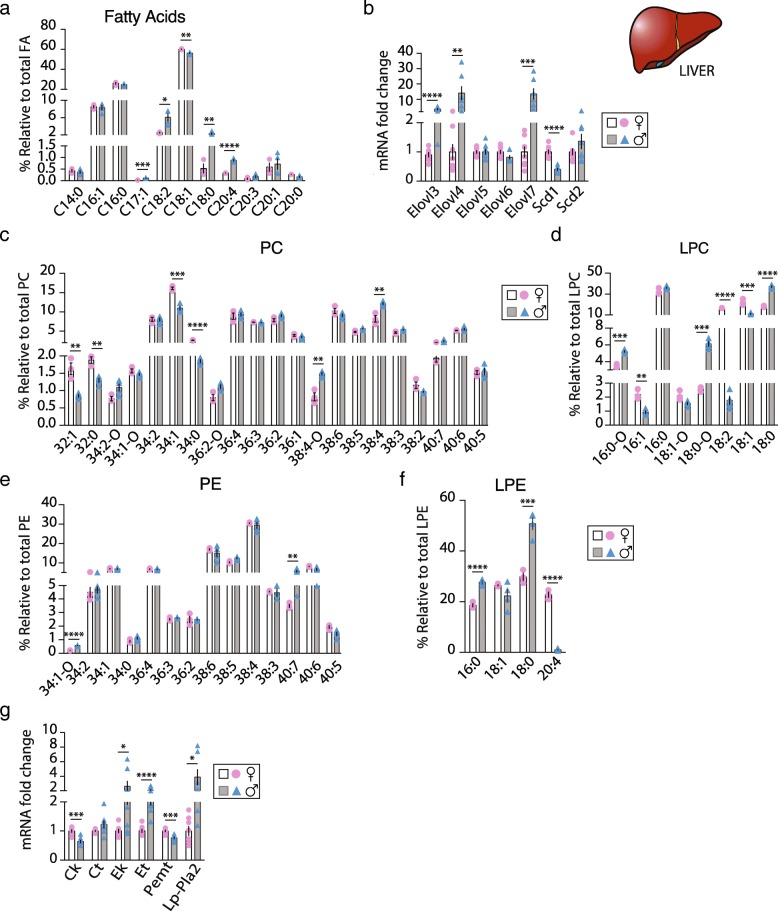
Sex-dependent FAs and PL profile in liver. Sex-dependent fatty acids (FAs) and phospholipid (PL) profile in liver of *ob/ob* F (♀ open bars and pink bullets) and M (♂ gray bars and blue triangles) mice. **a** Relative quantification of the most abundant FAs (*n* = 3–4) obtained by lipidomic analysis; **b** hepatic expression levels of elongases and desaturases genes; rRelative hepatic (**c**) phosphatidylcholine (PC), **d** lysophosphatidylcholine (LPC), **e** phosphatidylethanolamine (PE) and **f** lysophospatidylethanolamine (LPE) species content (*n* = 3–4); and **g** relative mRNA expression level of the PL synthesis pathway genes in F and M mice (*n* = 7–8). Values are means ± sem; *, *P* < 0.05, **, *P* < 0.01, ***, *P* < 0.001 and ****, *P* < 0.0001 M vs F were considered significant. *Elovl* fatty acid elongase, *Scd* fatty acid desaturase*, Ck* choline kinase, *Ct* choline transferase*, Ek* ethanolamine kinase, *Et* ethanolamine transferase, *Pemt* phosphatidylethanolamine *N*-methyltransferase and *Lp-Pla2* Lipoprotein associated phospholipase A2

Dysfunctions in one of the metabolic pathways involved in synthesis, transport, or removal of FAs and TGs are the basis for the development of liver steatosis. On a regular chow diet, the liver is the main organ that contributes to lipid production. To investigate whether F and M livers present different molecular species in their FA and PL profiles, a lipidomic analysis was carried out on total lipid extract from F and M livers. The proportion of C16, C18, and C20 FAs chains was similar between sexes (Additional file 2: Figure S1C) GC-FID identified a total of 11 FAs, with oleic acid (C18:1) > palmitic acid (C16:0) > palmitoleic acid (C16:1) > linoleic acid (C18:2) being the most abundant relative to the total amount of FAs in both F and M (Fig. 3a). The reduced multi-dimension plot (tSNE) of the FAs classes identified a high level of FAs homogeneity in the F group, while within the M group FAs classes were heterogenous (Additional file 3: Figure S2A). F livers had a higher relative amount of C18:1 (60.1% vs 56.3%, *p* < 0.01) whereas relative C18:2 > C18:0 > C20:4 > C17:1 content was higher in M compared to F (6.1% vs 2.7%, 2.3% vs 0.7%, 0.3 vs 0.9%, and 0.11% vs 0.03%, respectively, *p* ≤ 0.01) (Fig. 3a). To explore the mechanisms behind these sex-related FA profiles, the mRNA expression levels of genes involved in the elongation (*Elovl3*, *Elovl4*, *Elovl5*, *Elovl6*, *Elovl7*) and desaturation (*Scd1*, *Scd2*) of FAs were measured. M showed a higher expression level of *Elovl3*, *Elovl4*, and *Elovl7* (Fig. 3b). On the other hand, *Scd1* expression was significantly lower, thereby supporting a lower C18:1 relative content in M (Fig. 3a, b). Further supporting these results, the ratio C18:0/C16:0, a marker of elongase activity, was five times higher; whereas the C18:1/C18:0 ratio, indicative of desaturation, was six times reduced in M compared to F (Table 2). Finally, C16:0/C18:2 ratio, a marker of hepatic DNL activity, was 2.4 times higher in F than M. Moreover, the saturated FA (SFA) relative content was similar between sexes, but M showed respectively lower and higher relaive amount of mono-saturated FA (MUFA) poly-unsaturated FA (PUFA) compared to F. Therefore, MUFA/PUFA ratio was 2.3 times lower and PUFA/SFA 3 times higher in M (Table 2).

**Table 2:** Fatty acid profile (FAME measured by GC-FID) in liver, gAT and iAT

	Fatty acids	F	M		*P* values
LIVER	Total C16	34.5 ± 0.8	32.9 ± 0.3		ns
	Total C18	63.6 ± 0.3	64.7 ± 0.6		ns
	Total C20	1.2 ± 0.4	1.8 ± 0.3		ns
	C18:0/C16:0	0.02 ± 0.01	0.09 ± 0.01	**	*P* < 0.01
	C18:1/C18:0	146 ± 46	25 ± 3	*	*P* < 0.05
	C16:0/C18:2	10.2 ± 0.8	4.3 ± 0.6	**	*P* < 0.01
	Total SFA (%)	27.2 ± 0.4	27.5 ± 0.5		ns
	Total MUFA (%)	69.3 ± 0.2	65.4 ± 1.2	*	*P* < 0.05
	Total PUFA (%)	3.0 ± 0.3	7.2 ± 0.8	**	*P* < 0.01
	MUFA/SFA	2.5 ± 0.0	2.4 ± 0.1		ns
	PUFA/SFA	0.1 ± 0.0	0.3 ± 0.0	**	*P* < 0.01
	MUFA/PUFA	22.4 ± 1.9	9.6 ± 1.3	**	*P* < 0.01
gAT	Total C16	53.2 ± 3.6	43.8 ± 0.8	*	*P* < 0.05
	Total C18	45.9 ± 3.4	55.2 ± 0.9	*	*P* < 0.05
	C16:1/C16:0	0.46 ± 0.03	0.35 ± 0.02	*	*P* < 0.05
	Total SFA (%)	37.1 ± 3.2	33.3 ± 0.4		ns
	Total MUFA (%)	45.5 ± 1.2	45.2 ± 1.5		ns
	Total PUFA (%)	18.8 ± 0.4	21.3 ± 1.6		ns
	MUFA/SFA	1.3 ± 0.1	1.4 ± 0.1		ns
	PUFA/SFA	0.6 ± 0.0	0.6 ± 0.1		ns
	MUFA/PUFA	2.5 ± 0.1	2.2 ± 0.2		ns
iAT	Total C16	42.2 ± 4.4	35.3 ± 2.4		ns
	Total C18	55.0 ± 3.7	66.0 ± 2.3	*	*P* < 0.05
	C16:1/C16:0	0.49 ± 0.03	0.42 ± 0.08		ns
	Total SFA (%)	30.2 ± 3.4	26.0 ± 0.7		ns
	Total MUFA (%)	50.3 ± 2.2	48.0 ± 2.7		ns
	Total PUFA (%)	18.5 ± 0.8	28.5 ± 2.0	*	*P* ≤ 0.01
	MUFA/SFA	1.7 ± 0.2	1.8 ± 0.1		ns
	PUFA/SFA	0.6 ± 0.1	1.1 ± 0.1	*	*P* < 0.05
	MUFA/PUFA	2.7 ± 0.1	1.7 ± 0.2	*	*P* ≤ 0.01

Data are presented as mean ± sem, n = 3–4. Differences between sexes were determined by two-tailed Student’s *t* test with statistical significance determined using the Holm-Sidak method, with alpha = 5.000%. Each row was analyzed individually, without assuming a consistent SD. *, *P* < 0.05, **, *P* < 0.01, M vs F were considered significant. ns = not significant. *M* male, *F* female, *SFA* saturated fatty acid, *MUFA* monounsaturated fatty acid, *PUFA* polyunsaturated fatty acid, *gAT* perigonadal adipose tissue and *iAT* inguinal adipose tissue

To further investigate the potential role of female sex hormones on hepatic expression levels of the main genes that drive the FA pathways described above, we investigated the expression levels of several genes from the same lipid pathways in C57Bl/6J F and M wild-type (WT), estrogen receptor (ER) α knockout (KO), and ERβKO F mice. Gene expressions pattern within the WT (F and M) and ERαKO and ERβKO (F) mice groups was highly dependent of the sex and/or the loss of ERα or ERβ as shown in Additional file 4: Figure S4. *Srebp1c*, *Fas*, and *Acc1* expression levels were enhanced in ERβKO, *Srebp1c* in ERαKO F mice, and *Fas* in M WT, as compared to F WT, while *Hsl* and *Plin* expression levels were induced in ERβKO F only, with similar expression as M WT (Additional file 4: Figure S4A). *Elovl3*, *Elovl4*, and *Elovl7* hepatic expression levels were upregulated, and *Scd1* downregulated in M WT compared to F WT as observed in *ob*/*ob* model. ERαKO F mice displayed a higher expression level of *Elovl3* and *Elovl6* but downregulation of *Elov7* and *Scd2* as compared to WT F. On the contrary, ERβKO F mice had a higher expression level of *Elovl4* and lower of *Elovl5* compared to WT F (Additional file 4: Figure S4B).

In addition, we measured the expression level of these genes in liver of M and F WT mice on a high-fat induced obesity model. In HFD, most of the FAs are taken up by the liver from the circulation as opposed to chow diet where FAs are mainly synthetized by the liver. After uptake, non-esterified FAs are esterified into neutral lipid and packaged for secretion or stored. M had higher expression levels of *Fas* and *Acc1* and of all the elongases family compared to F and respectively higher and lower expression levels of *Scd1* and *Scd2* (Additional file 4: Figure S4C-D). Interestingly, M treated with E2 for 3 weeks rescued their expression level to the F level for all these genes except for *Elov3* and *scd2*. These data would support a transcriptional regulation of the FA synthesis by estrogens in liver and suggest that both ERs are involved in these regulations, as summarized in Fig. 5a. However, further studies will be necessary to unravel the mechanism by which sex hormones act as key regulators of lipid partitioning and hereby participate in the sexual dimorphism in obesity related diseases.

### Sex-dependent PL profile in liver

PLs comprise the most abundant class of membrane lipids and are a key component of the cellular membrane integrity that can behave as signaling molecules; thus, their levels are tightly regulated. We analyzed the two major hepatic PL classes phosphatidylcholine (PC) and phosphatidylethanolamine (PE), as well as lysophosphatidylcholine (LPC) and lysophosphatidylethanolamine (LPE), in order to identify a sex-specific signature of their molecular profiles. The reduced multi-dimension plot (tSNE) of these PL classes successfully cluster F and M groups (Additional file 3: Figure S2B). Most interestingly, reduced multi-dimension plot (tSNE) of each PL class identified, i.e., PC, LPC, PE, and LPE, clearly distinguished F and M specific PL profiles (Additional file 3: Figure S2C-D). Even though the proportion of each PC class (PC32 to PC40) did not differ between M and F, except for PC32 (Additional file 2: Figure S1D), among the 21 PC molecular species identified, six of them were statistically different between F and M when using a multiple *t* test corrected for multiple comparisons. PC34:1 (~ 13.5%), PC38:4 (~ 10.2%), PC38:6 (~ 9.7%), and PC36:4 (~ 9.1%) were the most abundant PC species in both sexes, compared to the rest of the PC species identified (Fig. 3c and Additional file 5: Table S1). The relative content of PC34:1 > PC34:0 > PC32:0 ≥ PC32:1 species to total PC were higher in F compared to M (16% vs 11%, 2.7% vs 1.9%, 1.9% vs 1.3% and 1.6% vs 0.9%, respectively, *p* < 0.01); in contrast, the relative content of PC38:4 > PC38:4-O species to total PC were higher in M than in F (12% vs 8% and 1.5% vs 0.8%, respectively, *p* < 0.01). Therefore, F PC profile showed enrichment of shorter acyl-chains as compared to M. In addition, M showed higher relative levels of the alkyl-substituted PC resulting in an overall higher level of total plasmalogen species within the PC class.

PC conversion by LP-PLA2 raises lysoPC (LPC) species. No differences into the LPC classes were observed (Additional file 2: Figure S1F); however, among the eight LPC molecular species identified, six of them were statistically different between F and M. LPC16:0 and LPC18:0 were the most present in both M and F livers (Fig. 3d and Additional file 5: Table S1). The percentage of LPC18:1 > LPC18:2 > LPC16:1 species was much higher in F compared to M (22% vs 11%, 17% vs 2% and 2.2% vs 0.9%, respectively, *p* < 0.0001); whereas the amount of LPC18:0 > LPC18:0-O ≥ LPC16:0-O species relative to total LPC was higher in M compared to F (37.2% vs 18.1%, 6.1% vs 2.6%, 5.2% vs 3.5%, respectively, *p* < 0.0001). These results indicate that M have higher proportion of saturated LPC and plasmalogen species whereas F show higher proportion of mono- and poly-unsaturated LPC species.

Among the PE classes, no difference between sexes were observed into the distribution (Additional file 2: Figure S1E) and 14 PE species were identified. When using a multiple *t* test corrected for multiple comparisons, only two of these were statistically different between sexes. However, the reduced multi-dimension plot (tSNE) of each PE class clearly distinguished F and M specific PE profiles (Additional file 3: Figure S2D). PE38 species were the most abundant PE species compared to the rest of the PE species identified in both sexes, total PE38 (~ 61%) with PE38:4 (~ 30%) > PE38:6 (~ 16%) > PE38:5 (~ 11%) > PE38:3 (~ 2.5%) and total PE40 (~ 13.5%) ≥ PE34 (~ 12.4%) ≥ PE36 (~ 11.7%). The proportion of PE40:7 > PE34:1-o were significantly higher in M compared to F (5.6% vs 3.5% and 0.6% vs 0.2%, respectively, *p* < 0.05) (Fig. 3e and Additional file 5: Table S1).

LPE class analysis showed clear sex-dependent distribution (Additional file 2: Figure S1D) with all LPE molecular species differently expressed between M and F (Additional file 2: Figure S1G). LPE species analysis showed that three out of the four species identified were drastically different between F and M, being more than ~ 20% higher in either F or M. Whereas, the proportion of LPE18:0 to total LPE was the most present in both sexes, in M, it was 70% higher than in F (51% vs 30%, respectively, *p* < 0.0001). In addition, M showed higher proportion of LPE16:0 than F (28% vs 18%, respectively, *p* < 0.0001). Interestingly, the percentage of LPE20:4 to total LPE was 22 times higher in F than in M (23% vs 1%, respectively, *p* < 0.0001) (Fig. 3f and Additional file 5: Table S1). Altogether, our data clearly reveal sex differences in hepatic PL and LPL composition, suggesting a sex-specific regulation of PL synthesis in mouse liver that may contribute to the sexual dimorphism observed during obesity.

To examine the mechanisms behind these sex-related PL pattern in mouse-livers, we measured the expression level of the main enzymes of the PL synthesis pathways (i.e., choline kinase (*Ck*), CTP:phosphocholine cytidylyltransferase (*Ct*), ethanolamine kinase (*Ek*), CTP:phosphoethanolamine cytidylytransferase (*Et*), PE N-methyltransferase (*Pemt*), and Lipoprotein-associated phospholipase A2 (*Lp*-*Pla2*)). In liver, PC species are synthesized via the choline pathway (*Ck* and *Ct*) or by methylation of PE via PE N-methyltransferase (*Pemt*) while PE species are synthesized by the ethanolamine pathway (*Ek* and *Et*). Interestingly, M showed a reduced *Ck* and *Pemt* mRNA expression level; whereas, the mRNA expression levels of *Ek*, *Et*, and *Lp-Pla2* were induced compared to F (Fig. 3g). These results suggest that in obese mouse livers, PC biosynthesis is favored in F, while PE and LPE biosynthesis are promoted in M. In line with these findings, M showed a decreased relative PC/PE ratio compared to F (1.73 vs 2.33, respectively, *p* < 0.01), that has been shown to adversely affect membrane integrity and result in liver damage [30]. Further supporting the sex-dependent enzyme activity of the PL synthesis, *Ck* and *Pemt* were less expressed, and *Ct* and *Lp-Pla2* were more expressed in WT M as well as in ERβKO F mice compared to WT F (Additional file 3: Figure S4E). ERαKO showed similar expression as WT F mice. On HFD, where most of the FAs are taken-up from circulation, M showed an overexpression of all genes of the PL pathway as compared to F but this expression was rescued to F level in M treated with estrogen (E2) for 3 weeks except for *Lp-Pla2* that stayed high (Additional file 4: Figure S4F). These data imply that the sex-dependent PL composition in liver could be partly driven by sex hormones both for synthesis and uptake as summarized in Fig. 5b.

### Sex-dependent FA species in gAT and iAT

**Fig. 4:**
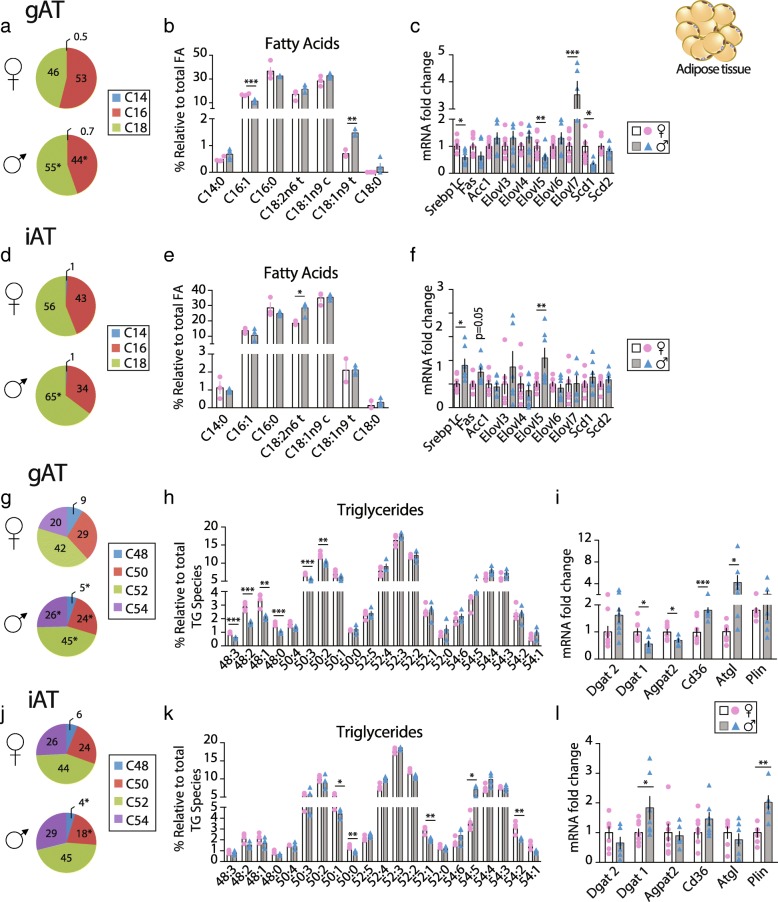
Sex-dependent FA and TG species in gAT and iAT. Sex-dependent gAT and iAT FA and TG profiles in ob/ob F (♀ - open bars and pink bullets) and M (♂ - gray bars and blue triangles) mice. Pie charts in **a** gAT and **d** iAT presenting the relative content of C14, C16 and C18 FA species; Relative quantification, in **b** gAT and **e** iAT, of the most abundant FAs found by lipidomic analysis (*n* = 4); Relative expression levels in **c** gAT and **f** iAT of de novo fatty acid synthesis genes (*n* = 7–9); Pie charts in **g** gAT and **j** iAT presenting the relative content of TG species; Relative quantification, in **h** gAT and **k** iAT, of the most abundant TGs found by lipidomic analysis (*n* = 4) and; Relative expression levels in **i** gAT and **l** iAT of the genes *Dgat2*, *Dgat1*, *Agpta2*, *Cd36*, *Atgl* and *Plin* (*n* = 7–9). Values are presented as mean ± sem; *, *P* < 0.05, **, *P* < 0.01 and ***, *P* < 0.001 M *vs* F were considered significant. Abbreviations: gAT: gonadal adipose tissue, iAT: inguinal adipose tissue, Elovl: fatty acid elongase, Scd: fatty acid desaturase, Srebp: sterol regulatory binding transcription factor, Fas: fatty acid synthase, Acc: acetyl-CoA carboxylase, Dgat: diacylglycerol O-acyltransferase, Agpat2: 1-acylglycerol-3-phosphate-O-acyltransferase 2, Cd36: cluster of differentiation 36, Atgl: adipose triglyceride lipase and Plin: perilipin

AT expansion is a key component of lipid homeostasis during overfeeding. Free FAs have been demonstrated as important mediators in the development of metaflammation in obesity. Adipocytes from VAT and SAT have different lipolytic and lipogenic properties. In our study, the ratio between total SAT and total VAT (SAT/VAT) was 32% higher in F (Table 1). Differences in fat distribution have been directly associated to different susceptibilities to metabolic diseases in human-obesity. However, little is known about sex-differences in lipid composition in these depots. Therefore, gAT and iAT were subjected to lipidomic analysis to characterize FAs composition and TG molecular species in F and M adipose depots. A relative amount of C16 FA species was significantly higher in gAT only and C18 lower in both gAT and iAT of F compared to M (Fig. 4a–d and Table 2). The reduced multi-dimension plot (tSNE) of the FAs classes identified high level of FAs homogeneity in the F group in the iAT as opposed to M, which showed high homogeneity in gAT (Additional file 6: Figure S3A).

GC-FID identified seven FAs in both fat pads (Fig. 4b and e) where palmitic acid (C16:0), palmitoleic acid (C16:1), oleic acid (C18:1n9c), and linoleic acid (C18:2n6t) were the most abundant FAs identified in all groups. In gAT, the proportion of C16:1 FA specie was higher in F than in M, in line with the higher C16:1/C16:0 ratio in F (Fig. 4b and Table 2). Interestingly, the relative content of the trans-FA C18:1n9t and C18:2n6t were lower in F in gAT and iAT, respectively (Fig. 4b, e). M and F showed comparable SFA and MUFA relative content in both adipose depots, but in iAT, M had higher relative PUFA content than F. Therefore, in iAT, as in liver, M presented a lower MUFA/PUFA ratio and higher PUFA/SFA than F (Table 2).

Lipid metabolism gene expression was studied to evaluate if the FA species revealed by lipidomic analysis were related to sex-dependent regulation at the transcriptional level. In gAT, de novo lipogenic gene *Srebp1c*, the desaturase *Scd1*, and the elongase *Elovl5* were significantly downregulated in M; as opposed to *Elovl7* that was upregulated compared to F (Fig. 4c). These findings support the lower C16:1 relative content and the higher C18 species content in M compared to F found in gAT. Oppositely, in iAT, *Srebp1c* and *Elovl5* expressions were upregulated in M compared to F in line with the longer chain FAs in M (Fig. 4f).

### Sex-dependent TG species in gAT and iAT

**Fig. 5:**
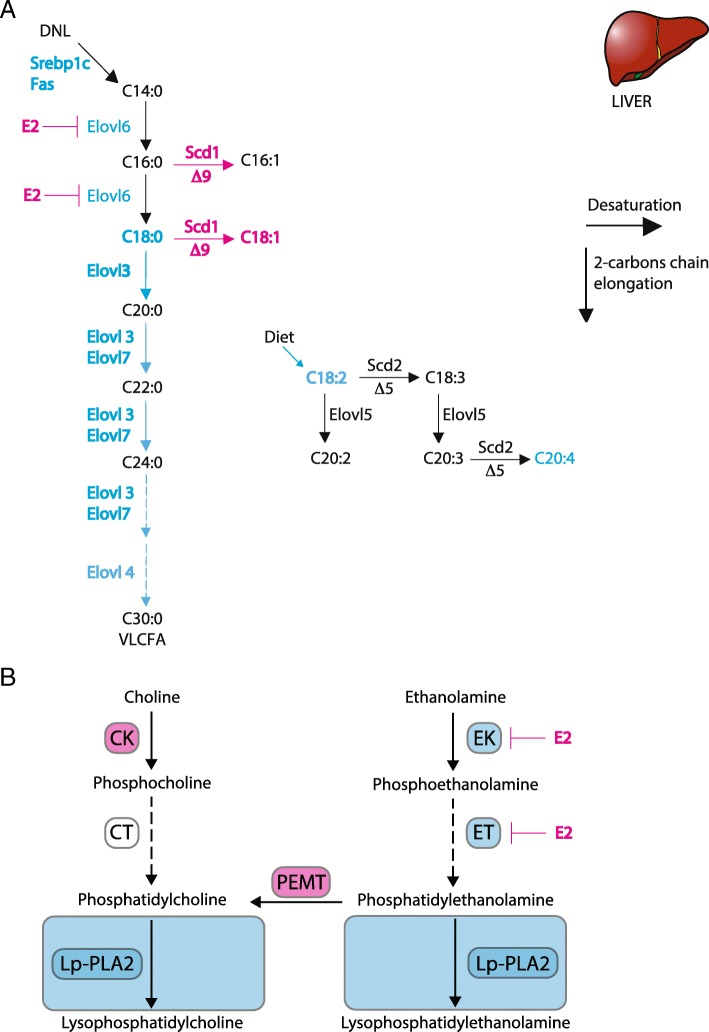
Sex differences in hepatic regulation of key genes of the FA and PL biosynthesis pathways. Graphical illustration of the transcriptional regulation of the **a** saturated, monounsaturated and polyunsaturated fatty acid biosynthesis pathways; **b** Phosphocholine/Lysophosphatidylcholine and Phosphoethanolamine/Lysophosphatidyl-ethanolamine synthesis pathways in F and M mouse liver. Blue denotes higher hepatic expression in male and pink in female mice; black shows no difference between sexes. Abbreviations: *Elovl*: fatty acid elongase, *Scd*: fatty acid desaturase, *Ck*: choline kinase, *Ct*: choline transferase*, Ek*: ethanolamine kinase, *Et*: ethanolamine transferase, *Pemt*: phosphatidylethanolamine *N*-methyltransferase and *Lp-Pla2*: Lipoprotein associated phospholipase A2

In AT, FAs undergo re-esterification into TGs and TG species have different metabolic properties; therefore, lipidomic analysis for TG molecular species in gAT and iAT have been performed. The reduced multi-dimension plot (tSNE) of the TG classes identified a high level of TG homogeneity in the F group in the gAT with one M showing similarity to F. The three other M showed high TGs homogeneity in gAT (Additional file 5: Figure S3B). In iAT, we found three clusters, with one F showing heterogeneity with the rest of the F group that was homogeneous in their TGs classes. Moreover, M did not show homogeneity in their TG classes. F gAT was, in percent, enriched in shorter chain TGs (C48 and C50) and had lower long chain TGs (C52 and C54) independently of their saturation degree, compared to M (Fig. 4g). 21 TG molecular species were identified by ESI-MS and MS/MS in all groups. Among them, in gAT six and in iAT five TG molecular species out of the 21 identified showed statistical differences between F and M when using a multiple *t* test corrected for multiple comparisons (Fig. 4h, k and Additional file 7: Table S2). In gAT, F displayed a higher percentage of TGs 50:2 > 50:3 > 48:1 ≥ 48:2 > 48:0 ≥ 48:3 compared to M (12.4% vs 10.2%, 7.0% vs 5.5%, 3.4% vs 2.1%, 2.9% vs 1.7%, 1.6% vs 1.0%, 1.0% vs 0.6%, respectively, *p* < 0.001). In iAT, F had a higher percentage of TGs 50:1 > 54:2 > 52:1 ≥ 50:0 (5.6% vs 4.4%; 3.2% vs 2.0%; 2.5% vs 2.1%; 1.4% vs 0.9%; F vs M respectively, *p* ≤0.01), whereas, TG54:5 was lower (3.6% vs 6.3%, respectively, *p* ≤0.01) in F than in M (Fig. 4h, k and Additional file 3: Table S2). These data indicate a sex-specific composition in TG species in both gAT and iAT. At the transcriptional level, the expression level of genes involved in the TGs synthesis showed significant differences between sexes especially in the gAT. In gAT, *Dgat1* and *AgPat2* were downregulated in M whereas *Cd36*, coding for the FAs transport in the adipocyte, and adipose triglyceride lipase (*Atgl*) were over expressed in M compared to F (Fig. 4i). In contrast, in iAT the mRNA level of *Dgat1* and *Plin* was higher in M with no differences in *Dgat2, AgPat2*, *Cd36* and *Atgl* expression levels (Fig. 4l). These data revealed a sex-specific TG species and synthesis in both adipose depots as presented in Fig. 5.

## Discussion

This study is an extensive characterization of a sex-specific regulation of lipid species composition in mouse liver, gAT and iAT, which may contribute to the sexual dimorphism in obesity. Despite differences in AT distribution as well as in liver and serum lipid composition between sexes, the biological role of lipid species in metabolic response is still unknown. In order to gain more insight into the sex-dependent lipid profile in obesity, our study focused on the characterization of the most abundant lipid classes and molecular species in liver (PC and PE) and in AT (TGs). This work unveils molecular signatures, which are markedly different between sexes. In addition, we showed that estrogen plays a key role in the regulation of the lipid synthesis in liver at the transcriptional level through both ERα and ERβ signaling pathways. This novel characterization could be of interest to identify sex-specific functional differences in the metabolic response observed in F and M obesity.

Although no differences in BW gain and food intake were found, physiological response to weight gain was sex dependent. F showed higher TF content and SAT/VAT ratio together with better insulin sensitivity and lower fasting insulin level compared to M. A higher plasma insulin concentration is positively correlated to the degree of AT inflammation [31]. The circulating levels of cytokines, which play a main role in the inflammation and IR in obesity, has been shown to be sex dependent [30–33]. In line with this, we found that resistin, which possesses pro-inflammatory effects and contributes to IR and inflammation [32, 33], was reduced in F. Oppositely, FGF21 and adiponectin, two anti-inflammatory adipokines with insulin sensitizing properties and negatively correlated to obesity, were higher in F. Conversely, M showed a high level of pro-inflammatory markers and crown-like structure in both AT compared to F, despite a lower proportion of total body fat. Therefore, our data show that M metabolic response to overfeeding a chow diet is altered compared to F which seems to be protected despite a higher total fat content.

**Fig. 6:**
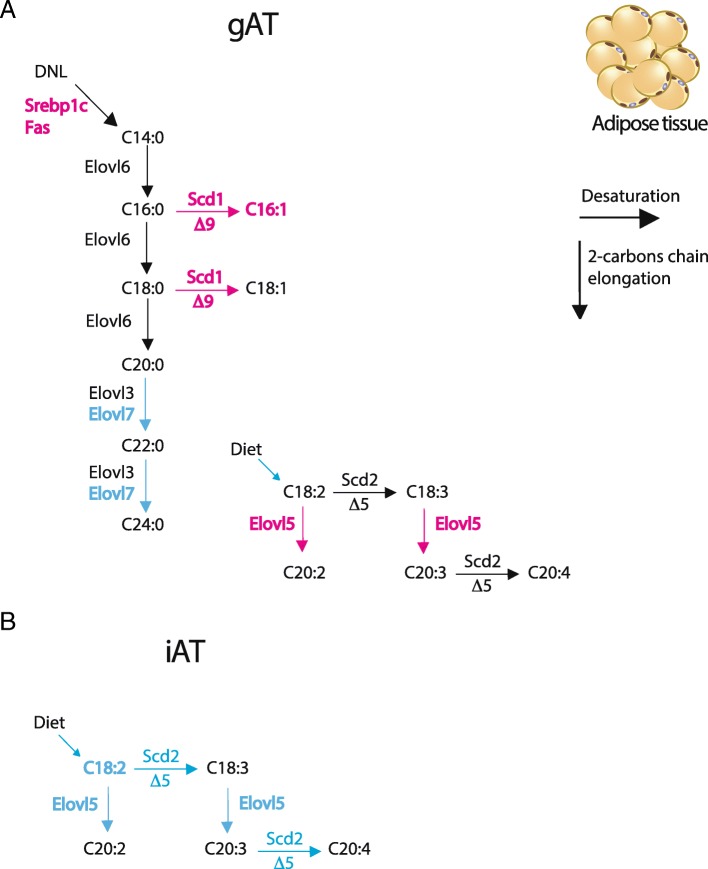
Sex differences in gAT and iAT regulation of genes of the FAs biosynthesis pathways. Graphical illustration of the transcriptional regulation of the saturated, monounsaturated and polyunsaturated fatty acid biosynthesis pathways in gAT (**a**) and iAT (**b**) F and M mouse. Blue denotes higher hepatic expression in M and pink in F mice; black shows no difference between sexes. Abbreviations: *Elovl*: fatty acid elongase and *Scd*: fatty acid desaturase

When diet contains low content of lipids (chow diet), FAs composition reflects, in most part, DNL pathway activity in the liver rather than FA uptake. De novo FAs composition is dependent of the activity of the elongase (*Elovl*) and the desaturase (*Scd)* enzyme family in the liver [27]. As proposed in the model in Fig. 6a, the DNL pathway in the liver for the synthesis of SFA, MUFA, and PUFA is sex dependent. Our results show that MUFAs biosynthesis was favored in F compared to M due to elevated desaturase Δ^9^D transcript level whereas long-chain PUFA (C20:4 and C18:2) and SFA (C18:0) were more abundant in M than in F. C18:0/C16:0 ratio, a marker of elongase activity in the liver, was ten times higher in M than F and has been recently identified as the most important factor that was correlated with the steatosis score in humans [34]. Our results suggest that liver steatosis observed in M livers could be the consequence of the sex-dependent regulation of key genes of the FAs pathways in the liver. Whether or not these genes are directly controlled by sex hormones remains to be discovered. However, previous data imply that *Elovl3* expression level in liver is under circadian variation in sexually mature males [35]. The authors conclude that androgens are essential for diurnal *Elovl3* expression in liver. Another study found that hepatic *Elovl3* gene expression level was induced 16 fold and *Scd1* induced 2 fold, in ERαKO F mice compared to WT F mice [36], in line with our study. F *ob*/*ob* mice treated for 3 weeks with E2 decreased the expression level of the hepatic lipogenic genes [37]. These data support a tight regulation of key genes of the lipid pathway by sex hormones at the transcriptional level that could initiate the sex dimorphism observed in metabolic disorders associated to obesity.

The biological implications of the changes in lipid composition are likely complex and difficult to predict simply on the basis of the FA or the PL compositions. The physiological outcomes of lipid composition depend on their locations (membrane, cytosolic, or nuclear) [38] and amounts [39]. For instance, arachidonic acid (AA, C20:4 n-6) is released from membrane PLs by phospholipase A2; cyclooxygenase then rapidly converts AA into a pro-inflammatory metabolite that accelerates the progression of hepatotoxicity [40, 41]. M livers had higher Δ^5^D activity, estimated by the 20:4/20:3 ratio, together with higher relative content of AA and a higher level of PC species containing AA. AA increases the risk for cardiovascular disease, and eicosanoids derived from AA may contribute to the development of inflammatory disorders [39]. In addition, increase of *n*6PUFA has been demonstrated to be linked to inflammation and, C18:2*n*-6trans FA is in higher proportion in M WAT than F. Although presently, we cannot directly relate sex-dependent FAs composition to inflammation, our results suggest that M intake of AA could be more detrimental than in F. Inflammatory response observed in obese M adipose depot could be the consequence of increased de novo synthesis of pro-inflammatory lipid species as compared to F.

The ratio between PC and PE reflects membrane integrity [30] and, a decrease in PC/PE ratio perturbs membrane integrity. This imbalance has been associated with liver failure [42]. M showed a decrease in the PC biosynthesis enzyme activity as summarized in Fig. 5b. Additionally, PC are required for very low-density lipoproteins (VLDL) secretion, as PC are the major lipids of the surface monolayer of the VLDL particles. A sex-dependent regulation of *Pemt* in the regulation of plasma high-density lipoproteins and VLDL has been demonstrated in mice [43], and estrogen has been shown to induce *Pemt* gene expression in human and mouse primary hepatocytes [44]. In addition, PEMT-deficient male mice had higher liver TG level as compared to WT-control males [45]. Additionally, diabetic patients with metabolic syndrome and cardiovascular diseases have higher Lp-PLA2 activity than those without the diseases [46, 47]. In line with these findings, *ob*/*ob* as well as WT M and ERβKO F mice showed lower hepatic *Pemt* together with higher *Lp-Pla2* expression levels compared to *ob/ob* F. On HFD, where most of the FAs are taken-up from circulation, M overexpressed, as compared to F, all genes of the PL, and E2 rescued this expression to the F level except for *Lp-Pla2* that stayed high. It is thus likely that M showed severe imbalance between PC and PE production due to PEMT and LP-PLA2 malfunction and that may imply liver dysfunctions (increased steatosis and TGs level) during obesity. Further studies should be performed to unravel the direct role of estrogens in these regulations.

AT and its stored lipid species are primarily derived from exogenous sources and endogenously synthesized via DNL, which are part of the lipid metabolism. In obesity, DNL capacity of adipocytes is substantially reduced and this may contribute to the associated metabolic perturbations. Studies have documented the possible connection between an increased DNL, particularly of palmitoleate (C16:1), in AT and systemic beneficial outcomes, such as an increased insulin sensitivity [48]. F gAT accumulated TAGs enriched in palmitic acid (C16:0) and myristic acid (C14:0), the direct products of DNL, with higher expression of the DNL genes (*Srebp1c* and *Fas*) and better insulin sensitivity than M. Increase of *n*6 PUFA levels has been demonstrated to be linked to inflammation and, C18:2*n*-6trans FA was found in higher proportion in M than F together with increased crown-like structures in M and a two-fold induction of *Tnfα* expression in both fat pads. Therefore, as in the liver, sex -specific response to obesity is likely to occur in adipose depots and could be different between iAT and gAT in M and F as suggested by recent studies [49, 50]. However, much remains to be learned about the factors that influence adipogenesis in the different depots and their contribution to metabolic health and diseases. Evidences raised from recent studies suggested that the differential regulation of FAs release and uptake in SAT and VAT modify their depot-specific metabolic properties [51, 52]. A possible role of sex hormones, especially estrogens, in white adipose function controlled by genes in development and pattern specification has been revealed in the last decade [50, 51].

We cannot exclude that in the *ob*/*ob* model with a C57BL/6 background, the absence of leptin production could be a limitation in the exploration of sex-dependent lipid profile in obesity and its translation to humans. However, recent studies have emphasized the need to develop gender appropriate medicine in lipid homeostasis especially in obesity and associated disorders [53–56]; and studies comparing different animal species agreed that the mouse is a suitable species for the study of human hepatic lipid metabolism [57, 58]. Although the transgenic model we used herein may not represent the exact lipid changes observed in humans, it has clearly demonstrated that alterations in lipid homeostasis in response to overfeeding are sex dependent. Central effect of leptin on food intake and lipid homeostasis has been largely explored and many groups showed the implication of leptin, insulin, and sex hormones in these regulations, at the central level [59–61] in both M and F [62]. However, to get more insight into the implication of sex hormones in these regulations, we also used two other models, (1) the high-fat diet induced obesity and (2) the ERKO mice for gene expression analysis. In both models, we observed sex-dependent and/or ER-dependent hepatic regulation in lipid gene expression that support our hypothesis. ERKO F mice brought crucial information on the potential implication of both ERs in these regulations, and WT M treated with E2 indicate that E2 treatment in M mice drives gene expression pattern towards the F one. Further studies should be performed to unravel the mechanism by which estrogen can affect lipid distribution and composition in obesity.

Altogether these findings provide more evidence to understand the sex-dependent metabolic response towards obesity. In the current work, we demonstrate that there is a characteristic lipid molecular profile in obesity in each sex, and we propose that this may drive sexual dimorphism associated to metabolic dysfunctions between M and F. The functional analysis of such changes is important but not simple and merits a study in its own. More research is needed to understand the functional significance of each PL species in disease progression, to assess whether PL and LPL metabolisms represent a promising target for the sex-dependent treatment of obesity-associated diseases. This work characterizes sex-specific lipid molecular species with active roles in metabolic homeostasis and has paved the way for recognizing uncharted avenues for potential therapies. Our studies reveal a fine sex-specific regulation of hepatic PL composition and TGs synthesis in WAT in obese mice. Knowing the importance of the high regulation of PLs in the cell membranes, this investigation opens a new field to unravel if the sex dimorphism observed in both sexes and the concomitant obesity-associated diseases are due to alterations in the PL pathway. Moreover, these sex-dependent molecular signatures found in obesity raise new questions, such as whether pharmacological treatment of obesity modifies these lipid molecular signatures in a sex-specific manner and whether these differences are driven by sex-specific hormones such as estrogens and/or androgens. Finally, we suggest that manipulating FA composition with diets and/or treatment could potentially be a new strategy in the treatment of metabolic diseases.

## Additional files

Additional file 1: **Table S3.** List of the primers used for RT-PCR and their sequence. (DOCX 19 kb)

Additional file 2: **Figure S1.** (A) F and (B) M representative photograph depicting the liver and the distribution of gAT and iAT (*n* = 7) and; representative histological sections immunostained for adipophylin in liver (*n* = 3–4); pie charts presenting the relative content of (C) FA classes; (D) PC classes; (E) PE classes; (F) LPC classes; (G) LPE classes. Abbreviations: FA: fatty acid, PC: phosphatidylcholine, PE: phosphatidylethanolamine, LPC: lysophosphatidylcholine and LPE: lysophosphatidylethanolamine. *, *P* < 0.05 M vs F were considered significant. (PDF 128080 kb)

Additional file 3: **Figure S2.** Sex-dependent hepatic FAs and phospholipid profile in liver of *ob/ob* mice. Relative hepatic t-SNE plot of (A) FAs; (B) phospholipids; (C) phosphatidylcholine (PC) and lysophosphatidylcholine (LPC); and (D) phosphatidylethanolamine (PE) and lysophospatidylethanolamine (LPE) species clusters in liver of *ob/ob* F (♀ pink bullets, *n* = 3) and M (♂ blue triangles, *n* = 4) mice. (PDF 168 kb)

Additional file 4: **Figure S4.** Hepatic gene expression levels in F WT (♀ pink bullets), M WT (♂ blue triangles), F ERαKO (♀ pink open diamonds) and F ERβKO (♀ pink filled diamonds) mice (*n* = 3–6) of (A) lipogenic pathway; (B) elongases and desaturases and (E) PL pathway. Hepatic gene expression levels in HFD fed mice (*n* = 8): F WT (♀ pink bullets, black bar), M WT (♂ blue triangles, filled gray bar) and M WT treated with estrogen (E2) (♂ blue triangles, stripped bar) of (C) lipogenic pathway; (D) elongases and desaturases and (F) PL pathway. Values are expressed as mean ± sem. Differences between groups were determined by Multiple *t* tests corrected for multiple comparison. Statistical significance was determined using the Holm-Sidak method, with alpha = 5.000% in GraphPad Prism (San Diego, CA). Each row was analyzed individually, without assuming a consistent SD. *, *P* < 0.05, **, *P* < 0.01, ***, *P* < 0.001 and ****, *P* < 0.0001 M vs F; ^#^, *P* < 0.05, ^##^, *P* < 0.01, ^###^, *P* < 0.001 and ^####^, *P* < 0.0001 M HFD vs M HFD-E2 and ^&^, *P* < 0.05, ^&&^, *P* < 0.01, ^&&&^, *P* < 0.001 and ^&&&&^, *P* < 0.0001, F WT vs F ERKO were considered significant. (PDF 306 kb)

Additional file 5: **Table S1.** Most probable phospholipid species (FAME measure by GC-FID) identified in liver. Relative amount of phosphatidylcholine (PC), lysophosphatidylcholine (LPC), phosphatidylethanolamine (PE) and lysophosphatidylethanolamine (LPE) molecular species. Data are presented as mean ± sem, *n* = 3–4. *p < 0.05 male (M) vs female (F) mice; ns = not significant. (DOCX 21 kb)

Additional file 6: **Figure S3.** Sex-dependent FAs and TGs profile in gAT and iAT of *ob/ob* mice. Relative t-SNE plot of (A) FAs; (B) TGs clusters in gAT and iAT of *ob/ob* F (♀ pink bullets, n = 4) and M (♂ blue triangles, *n* = 4). Abbreviations: gAT: perigonadal adipose tissue and iAT: inguinal adipose tissue. (PDF 129 kb)

Additional file 7: **Table S2.** Triglyceride molecular species identified in fat depots by MS/MS. Data are presented as mean ± sem. *n* = 4. *p < 0.05 male (M) vs female (F) mice; ns: not significant. Abbreviations: TG: triglycerides; gAT: perigonadal adipose tissue and iAT: inguinal adipose tissue. (DOCX 24 kb)

## Abbreviations

Acc: Acetyl-CoA carboxylase
Agpat: 1-acylglycerol-3-phosphate O-acyltransferases
AT: Adipose tissue
Atgl: Adipose triglyceride lipase
BW: Body weight
Ccl: C-C motif chemokine ligand
Cd: Cluster of differentiation
Ck: Choline kinase
Clec4f: C-type lectin domain family 4 member F
Dgat: Diacylglycerol O-acyltransferase
Ek: Ethanolamine kinase
Elovl: Elongase
FAs: Fatty acids
Fas: Fatty-acid synthase
FGF: Fibroblast growth factor
gAT: Perigonadal adipose tissue
Hsl: Hormone sensitive lipase
iAT: Inguinal adipose tissue
IL: Interleukins
LPL: Lysophospholipids
Lp-Pla2: Et, Lipoprotein-associated phospholipase A2
MCP: Monocyte chemoattractant protein
MUFA: Monounsaturated fatty acids
OGTT: Oral glucose tolerance test
PC: Phosphatidylcholine
PE: Phosphatidylethanolamine
Pemt: PE N-methyltransferase
PL: Phospholipids
Plin: Perilipin
PUFA: Polyunsaturated fatty acids
SAT: Subcutaneous adipose tissue
Scd: Desaturase
SFA: Saturated fatty acids
Srebp: Sterol regulatory element-binding protein
TGs: Triglycerides
TNF: Tumor necrosis factor
VAT: Visceral adipose tissue
