# Supplementary material for: Sex-specific lipid molecular signatures in obesity-associated metabolic dysfunctions revealed by lipidomic characterization in ob/ob mouse
Source: Biol Sex Differ. 2019 Feb 26;10:11. doi: 10.1186/s13293-019-0225-y (PMC6390380; doi:10.1186/s13293-019-0225-y)
Supplement: Supplementary file 1 — Table S3. List of the primers used for RT-PCR and their sequence. (DOCX 19 kb) [file 13293_2019_225_MOESM1_ESM.docx]

**Table S3. List of the primer sequences used for q RT-PCR**

| **Gene Symbol** | **Primer** | **Forward primer** | **Reverse primer** |
| --- | --- | --- | --- |
| 36b4 | 36b4 | GCTTCATTGTGGGAGCAGAC | ATGGTGTTCTTGCCCATCAG |
| Tf2b | Gtf2b | GTTCTGCTCCAACCTTTGCCT | TGTGTAGCTGCCATCTGCACTT |
| b-actin | Actb | ATGGTGGGAATGGGTCAGAA | ATCTGGGTCATCTTTTCACG |
| Srebp1-c | Srebp1 | GCCATGGATTGCACATTTGA | GGCCCGGGAAGTCACTG |
| Fas | Fasn | GGAGGTGGTGATAGCCGGTAT | TGGGTAATCCATAGAGCCCAG |
| Acc1 | Acaca | TGACAGACTGATCGCAGAGAAAG | TGGAGAGCCCCACACACA |
| Dgat1 | Dgat1 | ACAGAGCAGATGGGGCTG | TGGGACCTGAGCCATCAT |
| Dgat2 | Dgat2 | CCCTGAAGAACCGCAAAGG | CCTCCTCCAAGATCACCTGCTT |
| Hsl | Lipe | GGAGCACTACAAACGCAACGA | TCGGCCACCGGTAAAGAG |
| Plin | Plin | GCTTGACCATCAGAACCAATTTT | GAATCTGCCCACGAGAAAGG |
| Cd36 | Cd36 | GCCAAGCTATTGCGACATGA | TCTCAATGTCCGAGACTTTTCAA |
| Atgl | Pnpla2 | TGATGACCACCCTTTCCAACA | GCAGAGTATAGGGCACCATCATG |
| Agpat2 | Agpat2 | GCACCGTGGATAACATGAGC | CCATTGTCGTTGCGTGTACC |
| Scd1 | Scd1 | TCCGCCACTCGCCTACA | ACTTTCCCAGTGCTGAGATCGA |
| Scd2 | Scd2 | AGTATTGCCACCCAGATGCT | GTGGGGCACGAACCTTTACT |
| Elovl3 | Elovl3 | ATGAATTTCTCACGCGGGTTA | GAGCTTACCCAGTACTCCTCCAA |
| Elovl4 | Elovl4 | CCGGCAGCGTCCTGAAC | GTCCAGGTCCAGCGATAGAACT |
| Elovl5 | Elovl5 | TTCGATGCGTCACTCAGTACCT | TGTCCAGGAGGAACCATCCTT |
| Elovl6 | Elovl6 | TCAGCAAAGCAGCCGAAC | AGCGACCATGTCTTTGTAGGAG |
| Elovl7 | Elovl7 | TATGCCCGTGTGTGTTGTGT | TGTCAGTCCAAGCCTTCTTTC |
| Ck | Chkb | CCGGCCGTTGAAAACTCAAG | TCCATACCATGGAAACGGGC |
| Ct | Pcyt1a | AAGGAAACTCCGGGTAGTGG | TGGCTGTCATTGTACCTCGG |
| Et | Pcyt2 | GGCAATGACATCACGCTGAC | TCAGAGGACATCTGGCTGCT |
| Ek | Etnk1 | TCAGTTTGCGTTGGCTTCTC | TGGTTAAAACGGACAACCGC |
| LpPla2 | Pla2g7 | CCTGGAGCTAGTGTTGTGTGAG | AGCGAGTCAGATCCTAGCAC |
| Tnfa | Tnfa | CCCTCACACTCAGATCATCTTCT | GCTACGACGTGGGCTACAG |
| Il1b | Il1beta | TGGGCCTCAAAGGAAAGAAT | CAGGCTTGTGCTGCTTGT |
| Il6 | Il6 | TAGTCCTTCCTACCCCAATTTCC | TTGGTCCTTAGCCACTCCTTC |
| F4/80 | Erm1 | CCCCAGTGTCCTTACAGAGTG | GTGCCCAGAGTGGATGTCT |
| Cd68 | Cd68 | TGCGGCTCCCTGTGTGT | TCTTCCTCTGTTCCTTGGGCTAT |
| Ccl7 | Ccl7 | GTGTCCCTGGGAAGCTGTTA | AGAAAGAACAGCGGTGAGGA |
| Clec4f | Clec4f | GAGGCCGAGCTGAACAGAG | TGTGAAGCCACCACAAAAAGAG |
| Mcp1 | Ccl2 | GCTGGAGAGCTACAAGAGGATCACC | TCCTTCTTGGGGTCAGCACAGAC |
