# Supplementary figures and images for: Sex-specific lipid molecular signatures in obesity-associated metabolic dysfunctions revealed by lipidomic characterization in ob/ob mouse
Source: Biol Sex Differ. 2019 Feb 26;10:11. doi: 10.1186/s13293-019-0225-y (PMC6390380; doi:10.1186/s13293-019-0225-y)

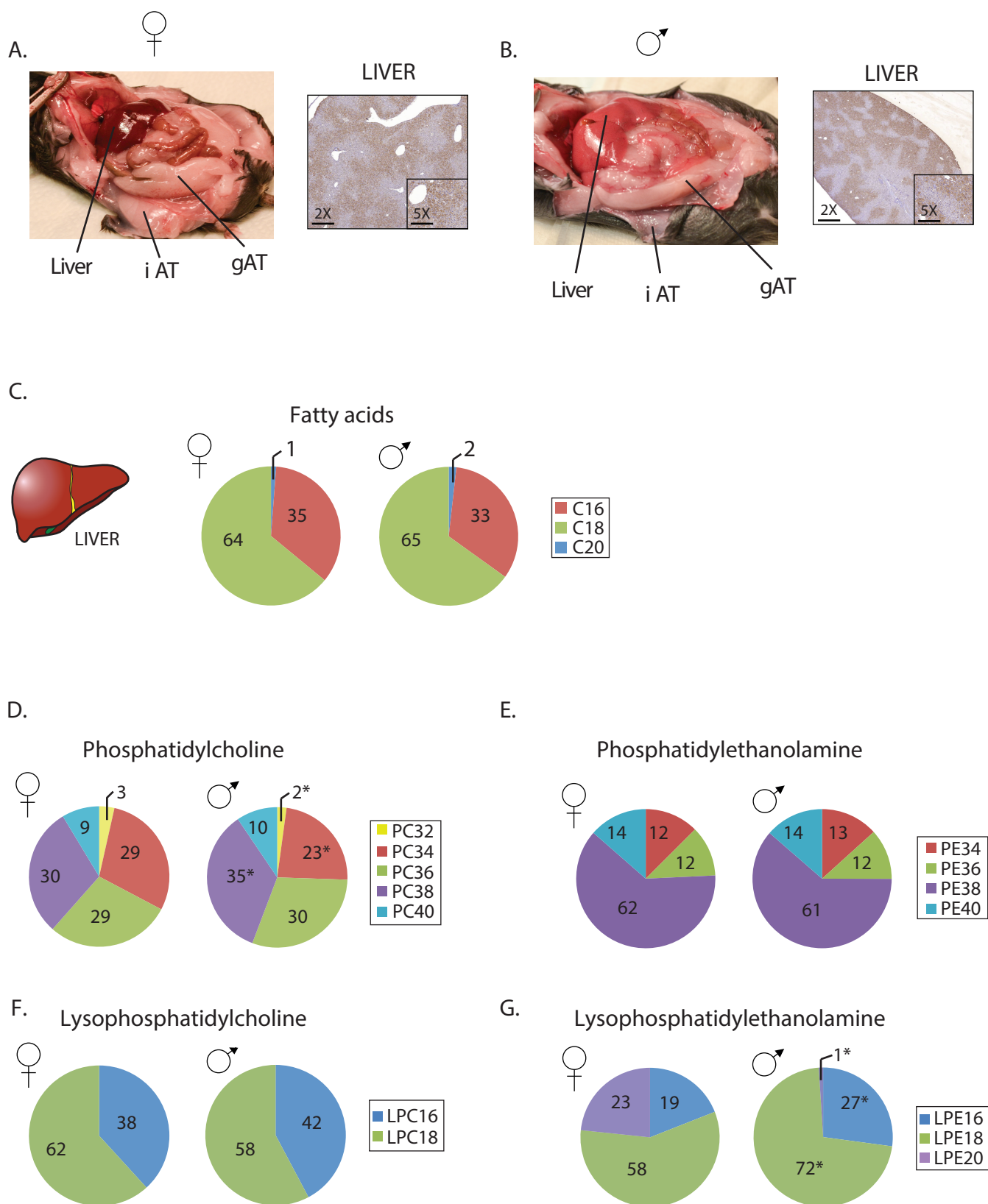

Suppl. Figure S1

Supplement: Supplementary file 2 — Figure S1. (A) F and (B) M representative photograph depicting the liver and the distribution of gAT and iAT (n = 7) and; representative histological sections immunostained for adipophylin in liver (n = 3–4); pie charts presenting the relative content of (C) FA classes; (D) PC classes; (E) PE classes; (F) LPC classes; (G) LPE classes. Abbreviations: FA: fatty acid, PC: phosphatidylcholine, PE: phosphatidylethanolamine, LPC: lysophosphatidylcholine and LPE: lysophosphatidylethanolamine. *, P < 0.05 M vs F were considered significant. (PDF 128080 kb) [file 13293_2019_225_MOESM2_ESM.pdf]

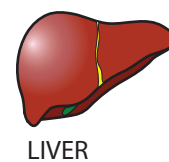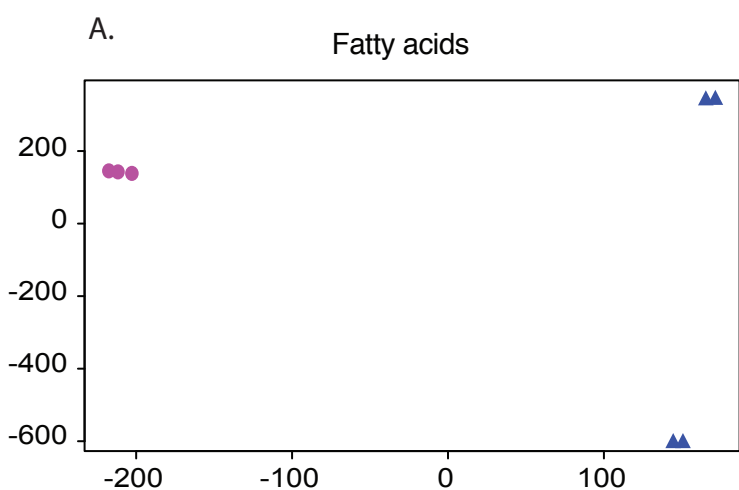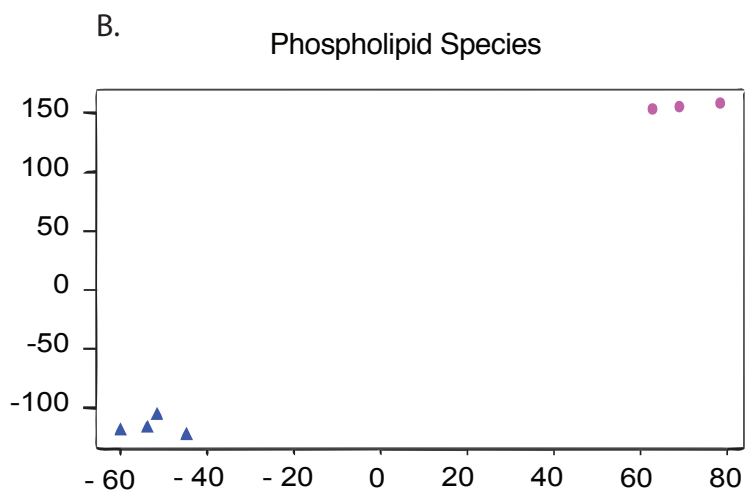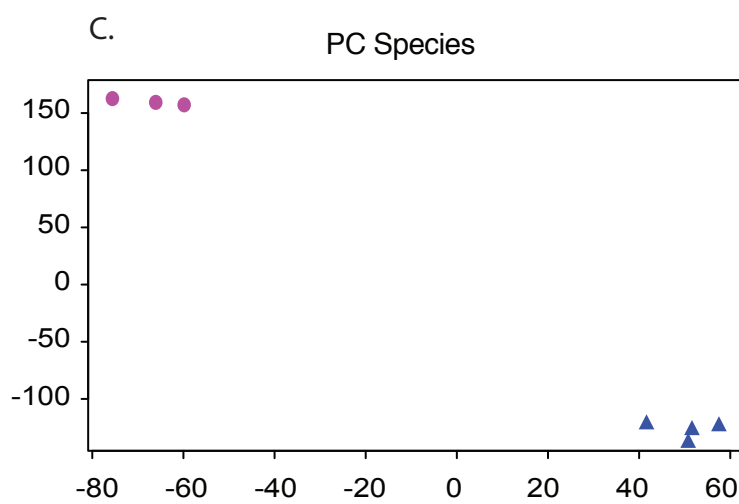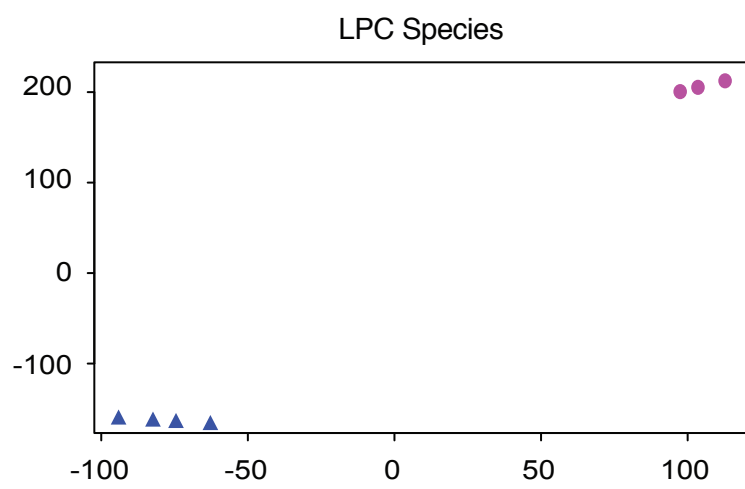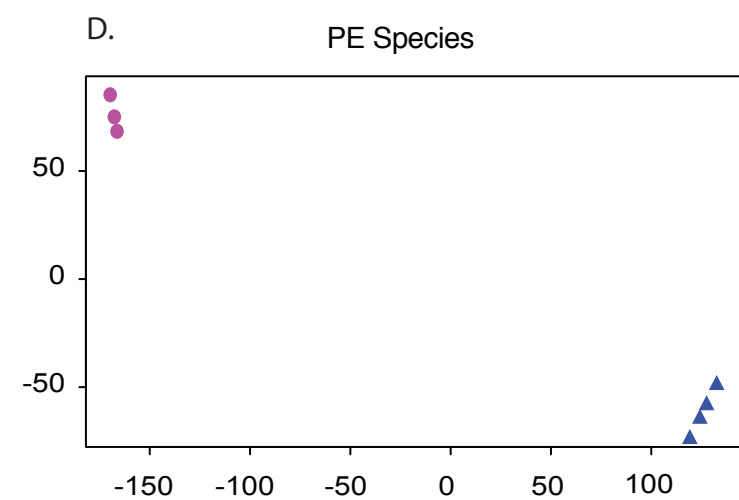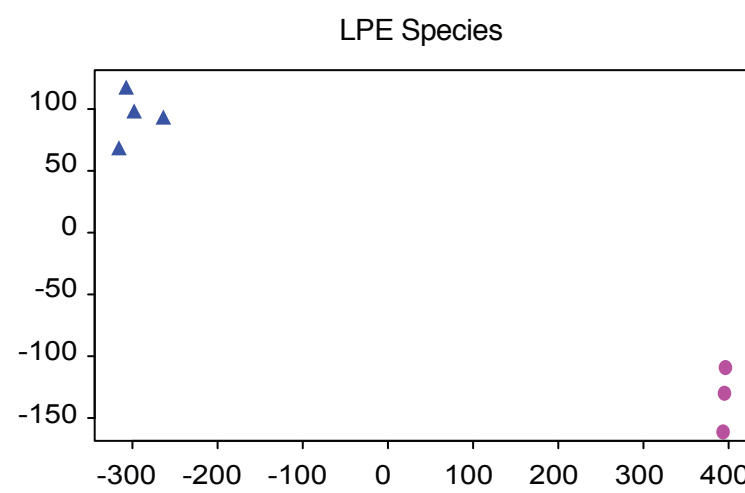

Supplement: Supplementary file 3 — Figure S2. Sex-dependent hepatic FAs and phospholipid profile in liver of ob/ob mice. Relative hepatic t-SNE plot of (A) FAs; (B) phospholipids; (C) phosphatidylcholine (PC) and lysophosphatidylcholine (LPC); and (D) phosphatidylethanolamine (PE) and lysophospatidylethanolamine (LPE) species clusters in liver of ob/ob F (♀ pink bullets, n = 3) and M (♂ blue triangles, n = 4) mice. (PDF 168 kb) [file 13293_2019_225_MOESM3_ESM.pdf]

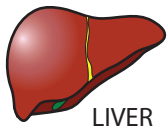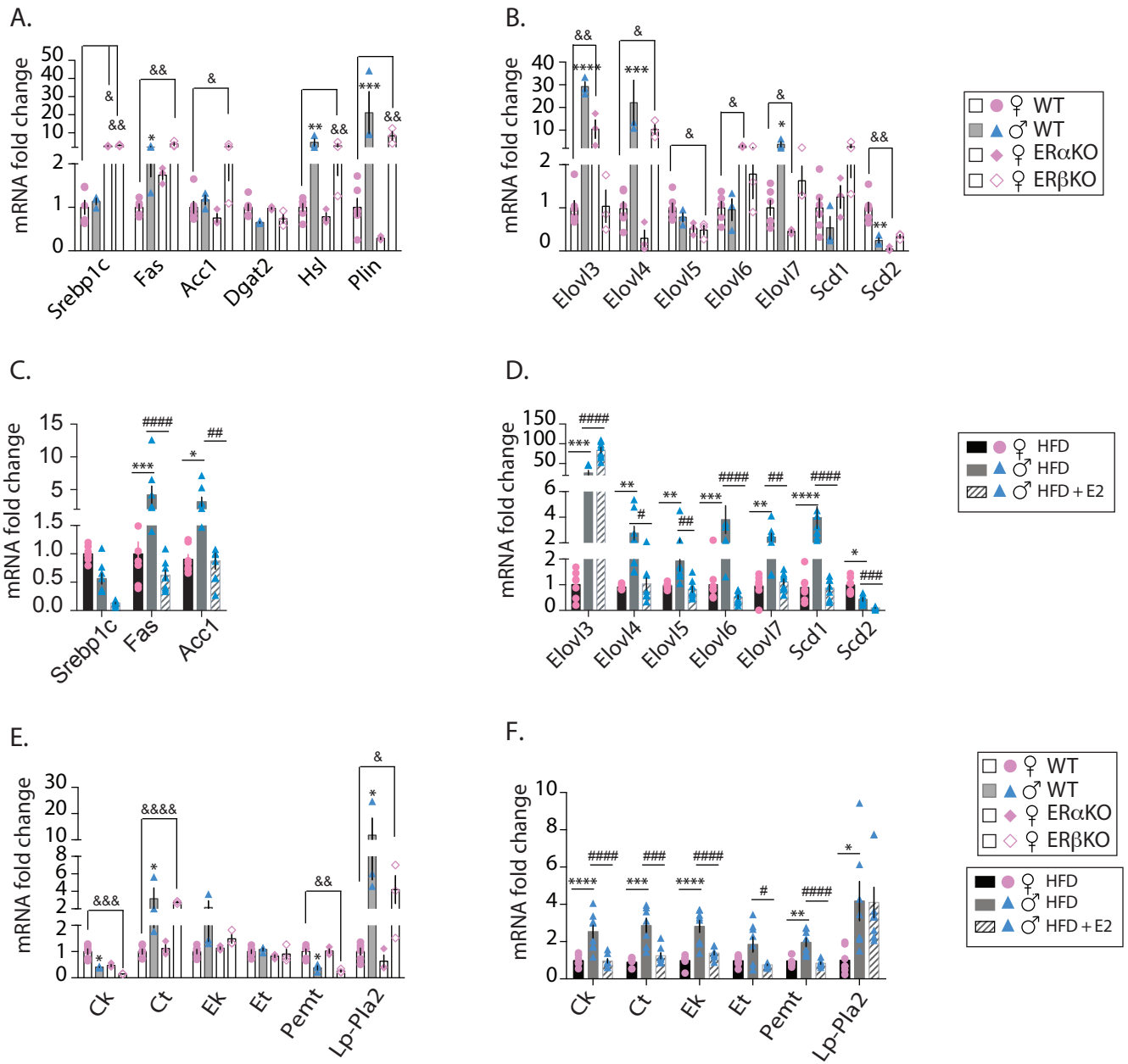

Suppl. Figure S4

Supplement: Supplementary file 4 — Figure S4. Hepatic gene expression levels in F WT (♀ pink bullets), M WT (♂ blue triangles), F ERαKO (♀ pink open diamonds) and F ERβKO (♀ pink filled diamonds) mice (n = 3–6) of (A) lipogenic pathway; (B) elongases and desaturases and (E) PL pathway. Hepatic gene expression levels in HFD fed mice (n = 8): F WT (♀ pink bullets, black bar), M WT (♂ blue triangles, filled gray bar) and M WT treated with estrogen (E2) (♂ blue triangles, stripped bar) of (C) lipogenic pathway; (D) elongases and desaturases and (F) PL pathway. Values are expressed as mean ± sem. Differences between groups were determined by Multiple t tests corrected for multiple comparison. Statistical significance was determined using the Holm-Sidak method, with alpha = 5.000% in GraphPad Prism (San Diego, CA). Each row was analyzed individually, without assuming a consistent SD. *, P < 0.05, **, P < 0.01, ***, P < 0.001 and ****, P < 0.0001 M vs F; #, P < 0.05, ##, P < 0.01, ###, P < 0.001 and ####, P < 0.0001 M HFD vs M HFD-E2 and &, P < 0.05, &&, P < 0.01, &&&, P < 0.001 and &&&&, P < 0.0001, F WT vs F ERKO were considered significant. (PDF 306 kb) [file 13293_2019_225_MOESM4_ESM.pdf]

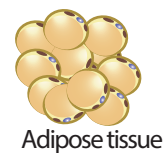

A.

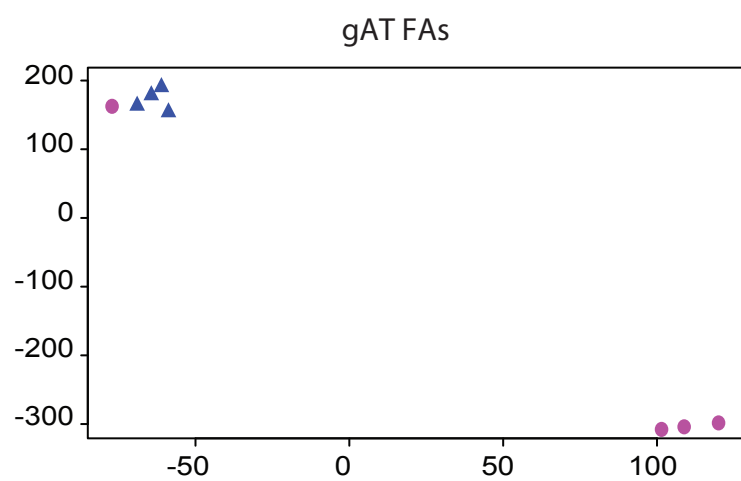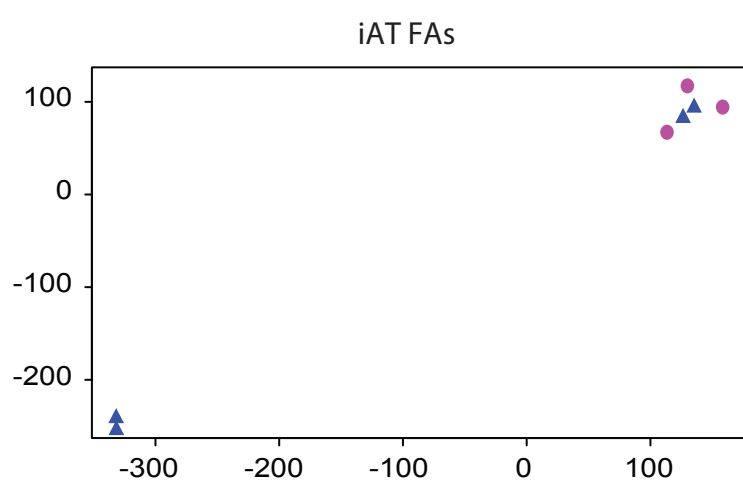

B.

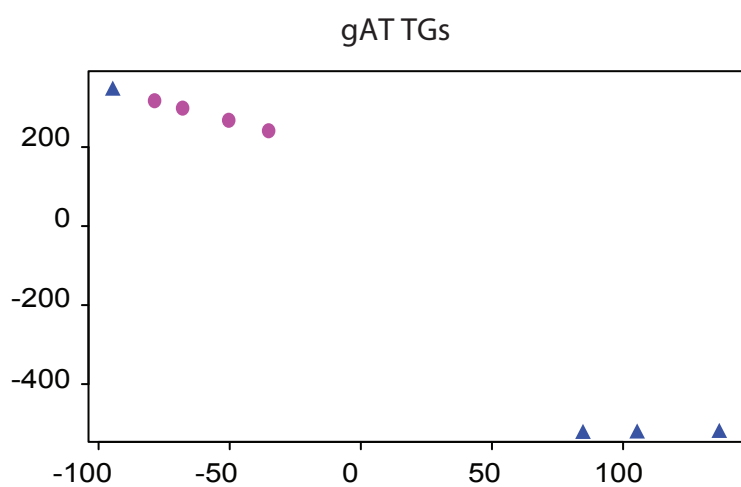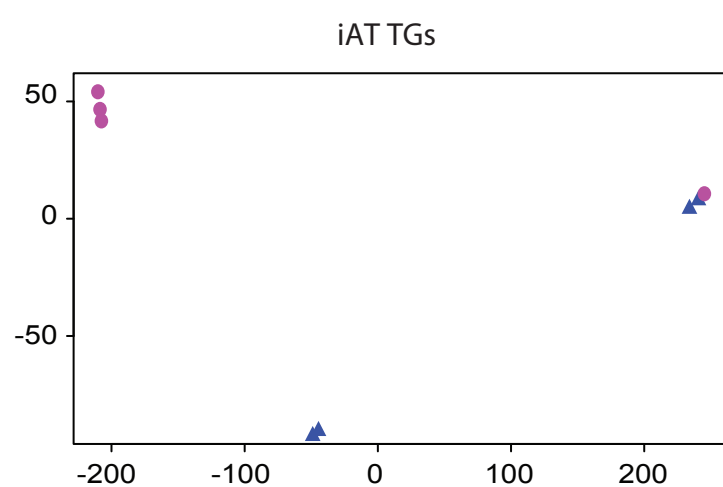

Supplement: Supplementary file 6 — Figure S3. Sex-dependent FAs and TGs profile in gAT and iAT of ob/ob mice. Relative t-SNE plot of (A) FAs; (B) TGs clusters in gAT and iAT of ob/ob F (♀ pink bullets, n = 4) and M (♂ blue triangles, n = 4). Abbreviations: gAT: perigonadal adipose tissue and iAT: inguinal adipose tissue. (PDF 129 kb) [file 13293_2019_225_MOESM6_ESM.pdf]
