# Supplementary material for: Sex-specific lipid molecular signatures in obesity-associated metabolic dysfunctions revealed by lipidomic characterization in ob/ob mouse
Source: Biol Sex Differ. 2019 Feb 26;10:11. doi: 10.1186/s13293-019-0225-y (PMC6390380; doi:10.1186/s13293-019-0225-y)
Supplement: Supplementary file 5 — Table S1. Most probable phospholipid species (FAME measure by GC-FID) identified in liver. Relative amount of phosphatidylcholine (PC), lysophosphatidylcholine (LPC), phosphatidylethanolamine (PE) and lysophosphatidylethanolamine (LPE) molecular species. Data are presented as mean ± sem, n = 3–4. *p < 0.05 male (M) vs female (F) mice; ns = not significant. (DOCX 21 kb) [file 13293_2019_225_MOESM5_ESM.docx]

Table S1: Most probable phospholipid species (FAME measure by GC-FID) identified in liver.

|  | PL | F | M |  | p values |
| --- | --- | --- | --- | --- | --- |
| **PC** | 32:1 | 1.63±0.17 | 0.85±0.04 | * | <0.01 |
|  | 32:0 | 1.88±0.09 | 1.30±0.06 | * | <0.01 |
|  | 34:2-O | 0.77±0.06 | 1.09±0.10 |  | =0.058 |
|  | 34:1-O / 33:1 | 1.57±0.06 | 1.47±0.03 |  | ns |
|  | 34:2 | 8.08±0.51 | 8.00±0.44 |  | ns |
|  | 34:1 | 16.14±0.35 | 10.97±0.48 | * | <0.001 |
|  | 34:0 | 2.66±0.04 | 1.85±0.05 | * | <0.001 |
|  | 36:2-O / 35:2 | 0.80±0.09 | 1.11±0.06 |  | ns |
|  | 36:4 | 8.89±0.81 | 9.37±0.41 |  | ns |
|  | 36:3 | 7.33±0.03 | 7.20±0.13 |  | ns |
|  | 36:2 | 7.86±0.38 | 9.03±0.30 |  | =0.06 |
|  | 36:1 | 3.92±0.23 | 3.52±0.22 |  | ns |
|  | 38:4-O / 37:4 | 0.82±0.12 | 1.49±0.04 | * | <0.01 |
|  | 38:6 | 10.22±0.67 | 9.12±0.35 |  | ns |
|  | 38:5 | 4.81±0.18 | 5.68±0.13 |  | ns |
|  | 38:4 | 8.19±0.85 | 12.16±0.29 | * | <0.01 |
|  | 38:3 | 4.64±0.20 | 5.36±0.15 |  | ns |
|  | 38:2 | 1.16±0.09 | 0.97±0.02 |  | ns |
|  | 40:7 | 1.93±0.11 | 2.42±0.17 |  | ns |
|  | 40:6 | 5.18±0.05 | 5.49±0.29 |  | ns |
|  | 40:5 | 1.52±0.06 | 1.55±0.10 |  | ns |
| **PE** | 34:1-O / 33:1 | 0.21±0.01 | 0.58±0.02 | * | <0.001 |
|  | 35:2-O / 34:2 | 4.50±0.37 | 4.67±0.23 |  | ns |
|  | 34:1 | 6.92±0.22 | 6.88±0.22 |  | ns |
|  | 34:0 | 0.90±0.08 | 1.12±0.08 |  | ns |
|  | 36:4 | 6.77±0.18 | 6.64±0.22 |  | ns |
|  | 36:3 | 2.51±0.08 | 2.61±0.03 |  | ns |
|  | 36:2 | 2.57±0.16 | 2.41±0.04 |  | ns |
|  | 38:6 | 16.96±0.42 | 14.95±1.89 |  | ns |
|  | 38:5 | 10.27±0.34 | 12.40±0.36 |  | ns |
|  | 38:4 | 30.47±0.21 | 29.35±1.61 |  | ns |
|  | 38:3 | 4.54±0.04 | 4.47±0.28 |  | ns |
|  | 40:7 | 3.50±0.12 | 5.57±0.59 | * | <0.05 |
|  | 40:6 | 8.14±0.19 | 6.58±0.62 |  | ns |
|  | 40:5 | 1.94±0.12 | 1.43±0.14 |  | ns |
| **LPC** | O-16:0 | 3.53±0.11 | 5.22±0.11 | * | <0.001 |
|  | 16:1 | 2.18±0.21 | 0.92±0.10 | * | <0.01 |
|  | 16:0 | 32.51±1.71 | 36.10±0.75 |  | ns |
|  | 17:1 or O-18:1 | 2.11±0.20 | 1.55±0.09 |  | ns |
|  | O-18:0 | 2.58±0.10 | 6.13±0.28 | * | <0.001 |
|  | 18:2 | 16.73±0.03 | 1.78±0.30 | * | <0.001 |
|  | 18:1 | 22.30±0.68 | 11.06±0.65 | * | <0.001 |
|  | 18:0 | 18.06±0.45 | 37.25±0.85 | * | <0.001 |
| **LPE** | 16:0 | 18.57±0.70 | 27.61±0.74 | * | <0.01 |
|  | 18:1 | 26.42±0.35 | 22.32±2.55 |  | ns |
|  | 18:0 | 29.83±1.51 | 50.81±2.39 | * | <0.01 |
|  | 20:4 | 22.79±1.06 | 1.04±0.24 | * | <0.001 |

Data are presented as mean ± sem, n=3-4. * p<0.05 male (M) *vs* female (F) mice;

ns= not significant. Abbreviations: PL: phospholipids, PC: phosphatidylcholine, PE: phosphatidylethanolamine, LPC: lysophosphatidylcholine and LPE: lysophosphatidylethanolamine
