# Supplementary material for: Sex-specific lipid molecular signatures in obesity-associated metabolic dysfunctions revealed by lipidomic characterization in ob/ob mouse
Source: Biol Sex Differ. 2019 Feb 26;10:11. doi: 10.1186/s13293-019-0225-y (PMC6390380; doi:10.1186/s13293-019-0225-y)
Supplement: Supplementary file 7 — Table S2. Triglyceride molecular species identified in fat depots by MS/MS. Data are presented as mean ± sem. n = 4. *p < 0.05 male (M) vs female (F) mice; ns: not significant. Abbreviations: TG: triglycerides; gAT: perigonadal adipose tissue and iAT: inguinal adipose tissue. (DOCX 24 kb) [file 13293_2019_225_MOESM7_ESM.docx]

Table S2: triglyceride species identified in gAT and iAT.

|  | TGs | F | M |  | p values |
| --- | --- | --- | --- | --- | --- |
| **gAT** | 48:3 | 0.98±0.03 | 0.61±0.03 | * | <0.001 |
|  | 48:2 | 2.87±0.15 | 1.68±0.07 | * | <0.001 |
|  | 48:1 | 3.40±0.24 | 2.08±0.09 | * | <0.01 |
|  | 48:0 | 1.60±0.06 | 1.03±0.05 | * | <0.001 |
|  | 50:4 | 1.66±0.08 | 1.32±0.07 | * | <0.01 |
|  | 50:3 | 6.98±0.15 | 5.54±0.19 | * | <0.001 |
|  | 50:2 | 12.35±0.38 | 10.19±0.29 | * | <0.01 |
|  | 50:1 | 7.33±0.39 | 5.98±0.32 | * | <0.05 |
|  | 50:0 | 1.16±0.02 | 1.20±0.15 |  | ns |
|  | 52:5 | 2.31±0.15 | 2.39±0.17 |  | ns |
|  | 52:4 | 7.79±0.61 | 9.09±0.71 |  | ns |
|  | 52:3 | 16.28±0.76 | 17.55±0.41 |  | ns |
|  | 52:2 | 11.90±0.23 | 12.17±0.76 |  | ns |
|  | 52:1 | 2.58±0.21 | 2.61±0.32 |  | ns |
|  | 52:0 | 0.82±0.07 | 1.20±0.33 |  | ns |
|  | 54:6 | 2.15±0.13 | 2.17±0.19 |  | ns |
|  | 54:5 | 3.41±0.35 | 4.82±0.60 |  | ns |
|  | 54:4 | 5.71±0.60 | 7.92±0.55 | * | <0.05 |
|  | 54:3 | 6.03±0.48 | 7.20±0.52 |  | ns |
|  | 54:2 | 2.39±0.28 | 2.34±0.29 |  | ns |
|  | 54:1 | 0.68±0.08 | 0.96±0.20 |  | ns |
|  | Relative TG48 | 8.9±0.4 | 5.4±0.1 | * | <0.001 |
|  | Relative TG50 | 29.5±0.5 | 24.2±0.5 | * | <0.001 |
|  | Relative TG52 | 41.6±1.1 | 45.0±0.2 | * | <0.05 |
|  | Relative TG54 | 19.8±1.4 | 25.4±0.8 | * | <0.05 |
| **iAT** | 48:3 | 0.91±0.07 | 0.82±0.07 |  | ns |
|  | 48:2 | 2.22±0.21 | 1.82±0.07 |  | ns |
|  | 48:1 | 2.20±0.20 | 1.65±0.07 |  | ns |
|  | 48:0 | 0.95±0.07 | 0.66±0.07 |  | ns |
|  | 50:4 | 1.50±0.02 | 1.41±0.07 |  | ns |
|  | 50:3 | 5.59±0.35 | 5.62±0.07 |  | ns |
|  | 50:2 | 10.24±0.38 | 8.93±0.07 |  | ns |
|  | 50:1 | 5.59±0.29 | 4.35±0.07 | * | ≤0.01 |
|  | 50:0 | 1.36±0.09 | 0.91±0.07 | * | ≤0.01 |
|  | 52:5 | 2.15±0.11 | 2.46±0.07 |  | ns |
|  | 52:4 | 7.81±0.40 | 10.02±0.07 |  | ns |
|  | 52:3 | 17.91±0.35 | 18.61±0.07 |  | ns |
|  | 52:2 | 12.14±0.27 | 10.74±0.07 |  | ns |
|  | 52:1 | 2.52±0.19 | 2.09±0.07 | * | <0.01 |
|  | 52:0 | 1.36±0.08 | 1.05±0.07 |  | ns |
|  | 54:6 | 1.84±0.20 | 2.40±0.07 |  | ns |
|  | 54:5 | 3.62±0.40 | 6.26±0.07 | * | ≤0.01 |
|  | 54:4 | 7.48±0.50 | 9.87±0.07 |  | ns |
|  | 54:3 | 7.68±0.11 | 7.36±0.07 |  | ns |
|  | 54:2 | 3.17±0.27 | 2.03±0.07 | * | ≤0.01 |
|  | 54:1 | 2.01±0.46 | 0.95±0.07 |  | ns |
|  | Relative TG48 | 6.3±0.5 | 4.95±0.7 |  | ns |
|  | Relative TG50 | 24.3±0.6 | 21.22±2.1 |  | ns |
|  | Relative TG52 | 43.9±0.6 | 44.96±0.4 |  | ns |
|  | Relative TG54 | 25.8±0.8 | 28.87±2.7 |  | ns |

Data are presented as mean ± sem. n=4. * p<0.05 male (M) *vs* female (F) mice; ns: not significant. Abbreviations: TG: triglycerides; gAT: perigonadal adipose tissue and iAT: inguinal adipose tissue.
